# Overview of the genus *Briareum* (Cnidaria, Octocorallia, Briareidae) in the Indo-Pacific, with the description of a new species

**DOI:** 10.3897/zookeys.557.6298

**Published:** 2016-01-28

**Authors:** Kaveh Samimi-Namin, Leen P. van Ofwegen

**Affiliations:** 1 Department of Marine Zoology, Naturalis Biodiversity Center, PO Box 9517, 2300 RA Leiden, the Netherlands

**Keywords:** Alcyonacea, Anthozoa, identification key, Oman Sea, Persian Gulf, sclerite variability, species range, synonymy

## Abstract

The status of Indo-Pacific *Briareum* species (Cnidaria, Octocorallia, Briareidae) is reviewed by presenting their sclerite features and habitus descriptions. Following the re-examination of type material, museum specimens and newly collected specimens, a species identification key is provided. The species distributions are discussed and updated distribution ranges are depicted. Moreover, a new taxon, *Briareum cylindrum* **sp. n.** is described and depicted, whereas *Briareum excavatum* (Nutting, 1911) is synonymised with *Briareum stechei* (Kükenthal, 1908). *Briareum hamrum* (Gohar, 1948) is recorded from the Persian Gulf and Oman Sea for the first time. Consequently, in total four *Briareum* species are recognized in the Indo-Pacific; *Briareum hamrum* from the western Indian Ocean, and *Briareum cylindrum* **sp. n.**, *Briareum stechei*, and *Briareum violaceum* from the central and eastern Indo-Pacific region.

## Introduction

*Briareum* Blainville, 1830 is the only genus in the family Briareidae with a wide distribution, occurring in both the Atlantic and the Indo-West Pacific (Fabricius and Alderslade 2001). It is zooxanthellate and therefore restricted to shallow, well-illuminated waters. It can be found in a wide range of habitats forming different colony shapes. The single Atlantic species, *Briareum asbestinum* (Pallas, 1766) has two main colony forms, encrusting and digitate (Bayer 1961; Bilewitch 2010). The Indo-Pacific species can form encrusting colonies, finger like lobes, or cylindrical branches, which may be hollow.

*Briareum* has unique morphological characteristics among octocoral genera. Corals of this genus are reasonably easy to recognize due to the characteristic shape and colour of their colonies and sclerites. The majority of the sclerites are spindles, some of them branched, with low or tall, spiny tubercles arranged in relative distinct girdles. The most basal layer generally includes multiple branched, reticulate and fused forms with very tall, complex tubercles. The medulla has magenta-coloured sclerites; the cortex may have magenta or colourless sclerites (Fabricius and Alderslade 2001). Only one species, *Briareum violaceum* (Quoy & Gaimard, 1833) has been recorded with magenta-coloured sclerites in both layers of the coenenchyme. In the literature, specimens with tall, deep magenta coloured calyces have usually been referred to *Pachyclavularia* Roule, 1908. Fabricius and Alderslade (2001) synonymized that genus with *Briareum*. The Indo-Pacific membranous and hollow-branched forms were referred to *Solenopodium* Kükenthal, 1916a. Bayer (1961) proposed *Solenopodium* as a junior synonym of *Briareum*. For details about the status of *Briareum* species refer to van Ofwegen (2015).

These morphological characters in *Briareum* species can show high variation in response to environmental factors such as depth, water motion, light, and predator damage (West 1997). For instance, high variation in colony and sclerite sizes, polyp density, egg size, and number of eggs has been reported for *Briareum asbestinum* along depth gradients in the Atlantic (West et al. 1993). These morphological variation and plasticity known from this genus together with inadequacy of descriptions in the literature has resulted in obscurity of the species characters, leading to misidentifications. This uncertainty in identification becomes obvious in the biochemistry and pharmacological studies in which the identification of source organisms is of great interest. It has been proven that *Briareum* offers extensive bioactive chemical compounds with antiviral, and antimicrobial properties (Chen et al. 2006; Wang et al. 2012; Yeh et al. 2012), and it is the most important source of briarane-type metabolites among the diterpenoids isolated from octocorals (Sung et al. 2002; Hong et al. 2012). In spite of *Briareum* being a valuable and an important source of biochemical compounds, the identifications of these species usually remains unsatisfactory and uncertain. In addition to their variation in shape and the lack of accurate morphological descriptions, the extent of molecular knowledge about different species is also limited. Although molecular records from the Indo-Pacific are rare, *Briareum* is distinctly recognized as one of the basal genera in the Octocorallia phylogeny (McFadden et al. 2006). The current records suggest the existence of at least three different species of *Briareum* across the Indo-Pacific region (McFadden et al. 2011, 2014; Miyazaki and Reimer 2014; GenBank (http://www.ncbi.nlm.nih.gov/genbank/). These data emphasize the need for further morphological and molecular knowledge about *Briareum* species across wider geographical areas.

**Figure 1.**
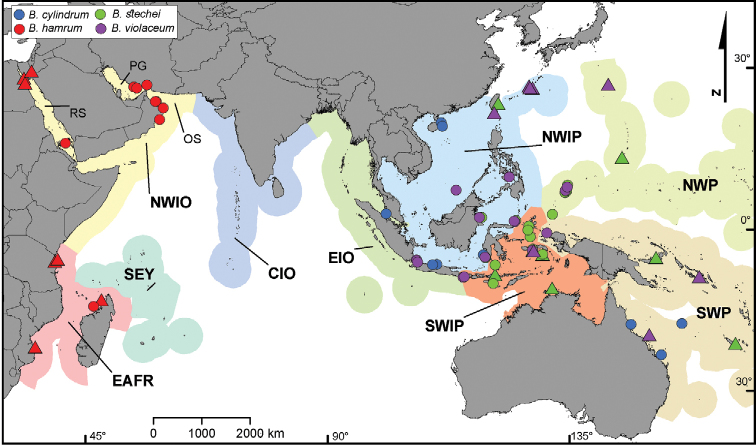
Distribution map of Indo-Pacific *Briareum* species based on: ● = examined material; ▲ = literature records. Colour shades on the background represent different marine regions. PG = Persian Gulf; OS = Oman Sea; RS = Red Sea; NWIO = North Western Indian Ocean; SEY = Seychelles; EAFR = East Africa; CIO = Central Indian Ocean; EIO = East Indian Ocean; SWIP = South West Indo-Pacific; NWIP = North West Indo-Pacific; NWP = North West Pacific; SWP = South West Pacific.

Here, the sclerite features and descriptions of *Briareum* species are presented based on the re-examination of type specimens, museum material, and newly collected material from the Indian Ocean and Indo-Pacific region, much of which is from the centre of maximum marine species richness, the Coral Triangle (Hoeksema 2007). An identification key to the presently recognized Indo-Pacific species is provided, a new taxon is described and two species are synonymised. Moreover, we show the variability of the sclerites among examined material and point out the difficulties, uncertainties and potential topics for further research. A distribution map of the examined material is also provided, together with all published species for the Indian Ocean and Indo-Pacific region (Figure 1). This study can be used in molecular and biochemical studies and may help coral researchers to identify *Briareum* material.

### Abbreviations

NBC
Naturalis Biodiversity Center, Leiden, The Netherlands; previously National Museum of Natural History (NNM); formerly Rijksmuseum van Natuurlijke Historie (RMNH)


OCDN/OPHG
 Numbers used by the Coral Reef Research Foundation, Palau 


RMNH
 Rijksmuseum van Natuurlijke Historie, currently NBC 


UNESCO-IOC
 United Nations Educational, Scientific and Cultural Organization- Intergovernmental Oceanographic Commission 


UNHAS
 Universitas Hasanuddin, Makassar, Indonesia 


ZMA
Zoological Museum Amsterdam, Amsterdam, The Netherlands 


ZMB
 Zoologisches Museum Berlin, Berlin, Germany 


## Material and methods

All studied material is deposited in the Naturalis Biodiversity Center. All *Briareum* specimens deposited in the RMNH coelenterate collection were examined, including misidentified material. Additional specimens collected by the Coral Reef Research Foundation, Palau, were also examined.

In order to identify the material, sclerites were obtained by dissolving the tissues in 10% sodium hypochlorite, followed by rinsing in fresh water. Due to variation in size and shape of the sclerites, it is recommended to use all parts of the colony. For example, missing calyces might result in finding shorter sclerites. For scanning electron microscopy (SEM), the sclerites were carefully rinsed with double-distilled water, dried at room temperature, were mounted on a stub with double-sided carbon tape, then coated with gold-palladium (AuPd), and examined using a Jeol 6480LV SEM operated at 10 kV.

## Morphological descriptions and systematic account

### Class Anthozoa Ehrenberg, 1831 Subclass Octocorallia Haeckel, 1866 Order Alcyonacea Lamouroux, 1812 Family Briareidae Blainville, 1830

#### Genus Briareum

Taxon classificationAnimaliaAlcyonaceaBriareidae

Blainville, 1830

Briareum Blainville, 1830: 484
Asbestia Nardo, 1845: 106
Pachyclavularia Roule, 1908: 165
Solenopodium Kükenthal, 1916a: 174

##### Diagnosis.

Colonies lobate, digitate or encrusting, normally with a whitish outer layer and magenta inner layer, but completely magenta or white colonies also occur. Polyps monomorphic, retractile, and without sclerites. Protruding false calyces appear in varying degrees of prominence or are not present at all. Surface layer with straight or curved spindles. Medulla with sclerites shaped like those of the surface layer but larger and coarser, and with additional branching sclerites, which can be fused. Zooxanthellate.

##### Distribution.

The genus has been recorded from the Caribbean and the Indo-Pacific (Red Sea, Persian Gulf, Oman Sea, Arabian Sea, Australia, Indonesia, Micronesia, Taiwan, and Bonin Islands).

### Type species

#### Briareum asbestinum

Taxon classificationAnimaliaAlcyonaceaBriareidae

(Pallas, 1766)

Alcyonium asbestinum Pallas, 1766: 344.
Briareum gorgonoideum Blainville, 1830: 484.
Ammothea polyanthes Duchassaing & Michelotti, 1860: 15, pl. 1 fig. 6.
Erythropodium marquesarum Kükenthal, 1916a: 173; 1919: 34 (Marquesas-Islands, Caribbean)
Briareum asbestinum Kükenthal, 1916b: 469, figs F–H, pl. 23 figs 1–7; Verseveldt 1940: 9, figs 2–4; Bayer 1961: 62, fig. 11; Bilewitch et al. 2010: 93.

##### Distribution.

Caribbean, Gulf of Mexico.

### Key to the Indo-Pacific *Briareum* species

**Table:** 

1	Coenenchymal spindles up to 0.45 mm long with prominent, sparsely set tubercles	***Briareum hamrum***
–	Coenenchymal spindles longer than 0.45 mm long with low, closely set tubercles	**2**
2	Many cylinders present in coenenchyme, with dense tuberculation	***Briareum cylindrum* sp. n.**
–	Only spindles present in coenenchyme	**3**
3	Many spindles with pointed ends in coenenchyme, all sclerites magenta	***Briareum violaceum***
–	Many spindles with blunt ends in coenenchyme, sclerites magenta and colourless	***Briareum stechei***

### Briareum cylindrum sp. n.

Taxon classificationAnimaliaAlcyonaceaBriareidae

http://zoobank.org/CDFC1779-62C2-4F27-943C-329D9F28BC3C

**Figure 2.**
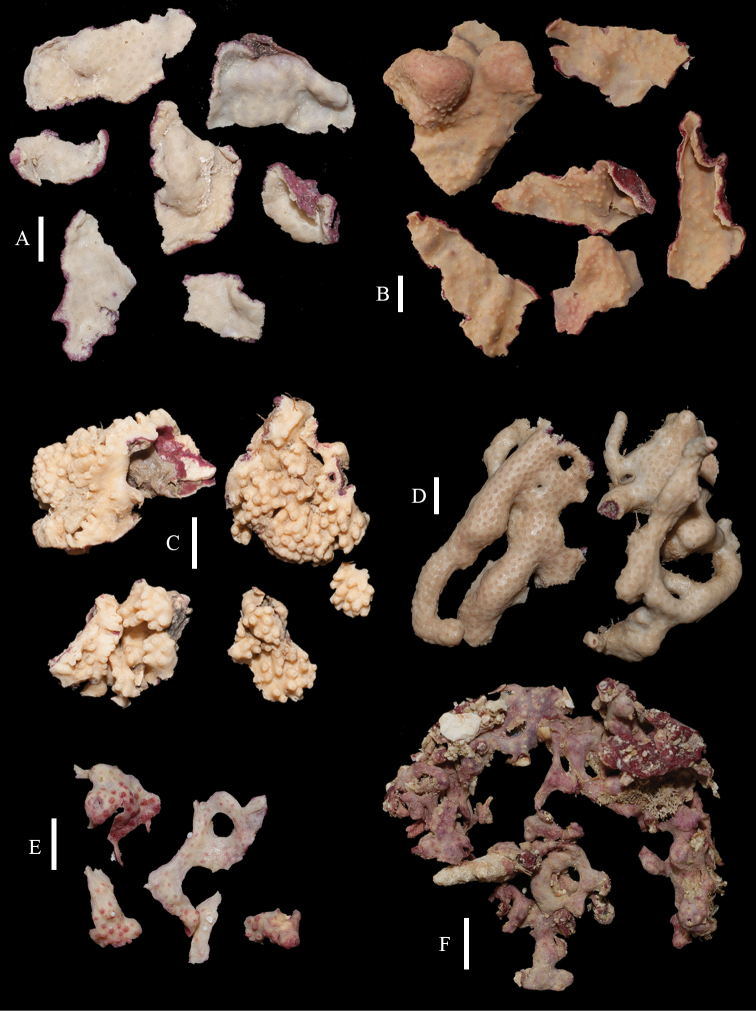
Colonies of *Briareum*: **A–D** *Briareum cylindrum* **A** RMNH Coel. 34193 (holotype) **B** RMNH Coel. 13747 **C** RMNH Coel. 32569 **D** RMNH Coel. 41443 **E–F** *Briareum hamrum* **E** RMNH Coel. 6809 **F** RMNH Coel. 41407. Scale bars: 1 cm.

**Figure 3.**
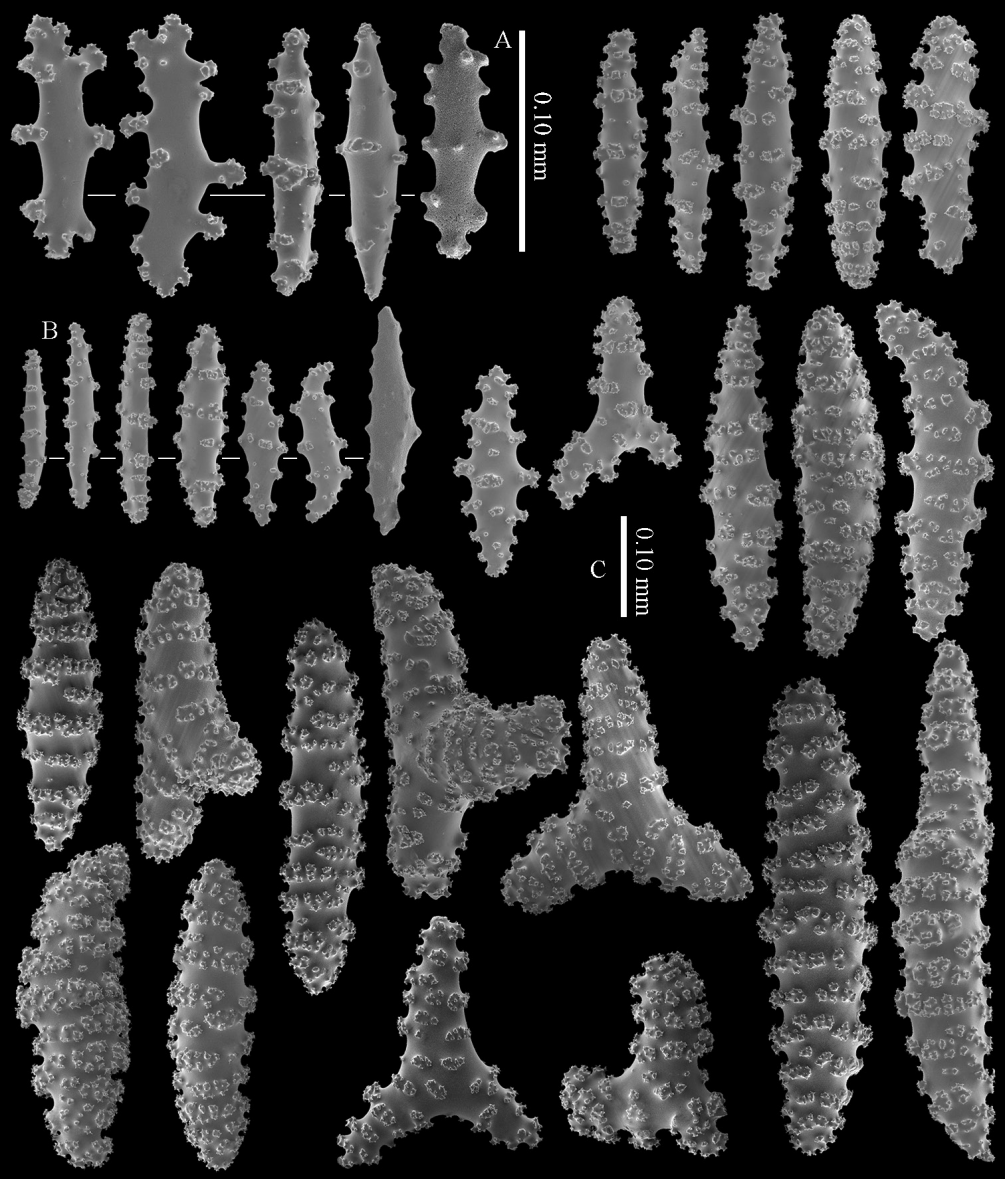
*Briareum cylindrum* sp. n., holotype, RMNH Coel. 34193 **A–B** sclerites of top calyx **C** cortex sclerites. Scale bar of **C** also applies to **B**.

**Figure 4.**
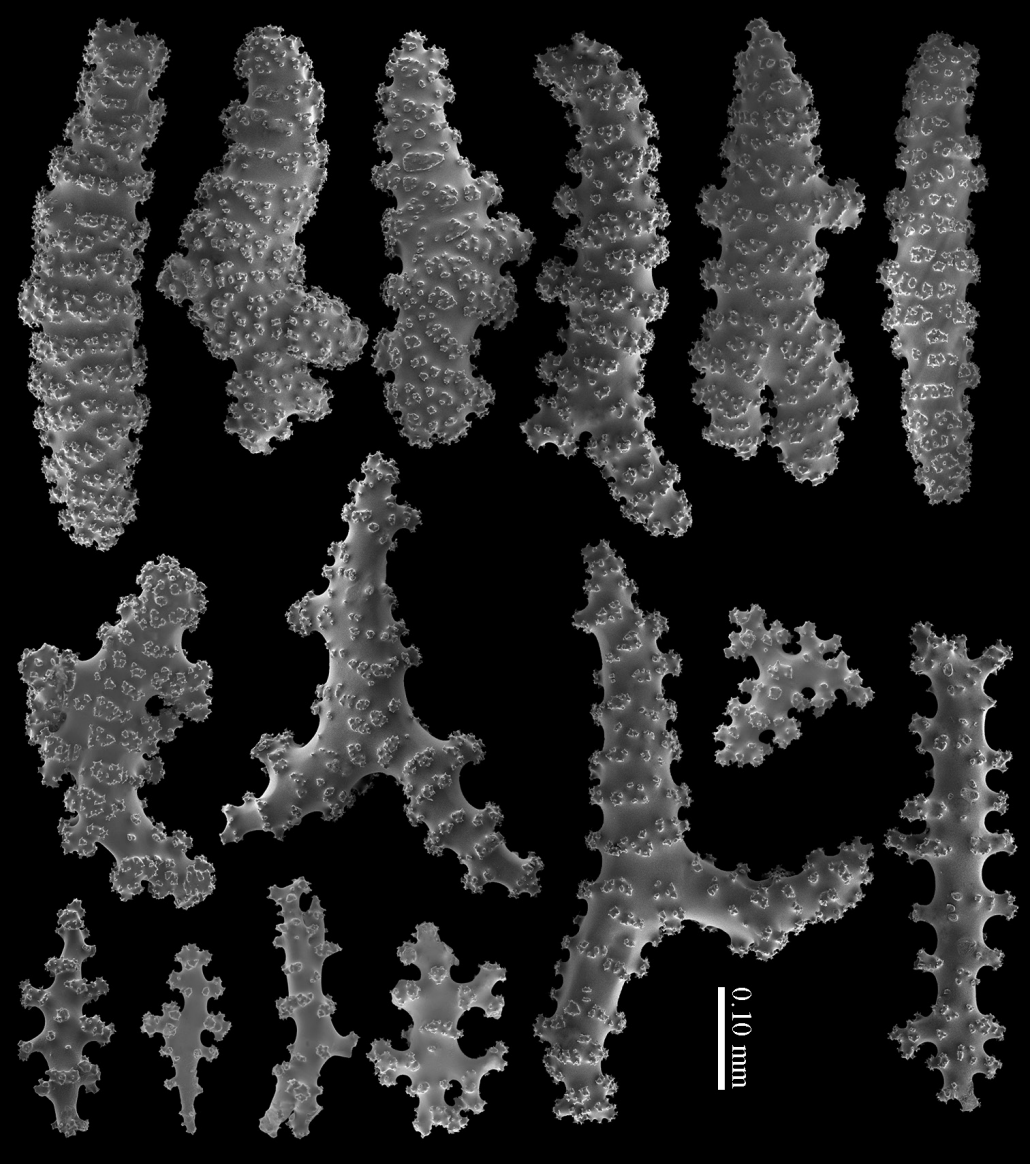
*Briareum cylindrum* sp. n., holotype, RMNH Coel. 34193, medullar sclerites.

**Figure 5.**
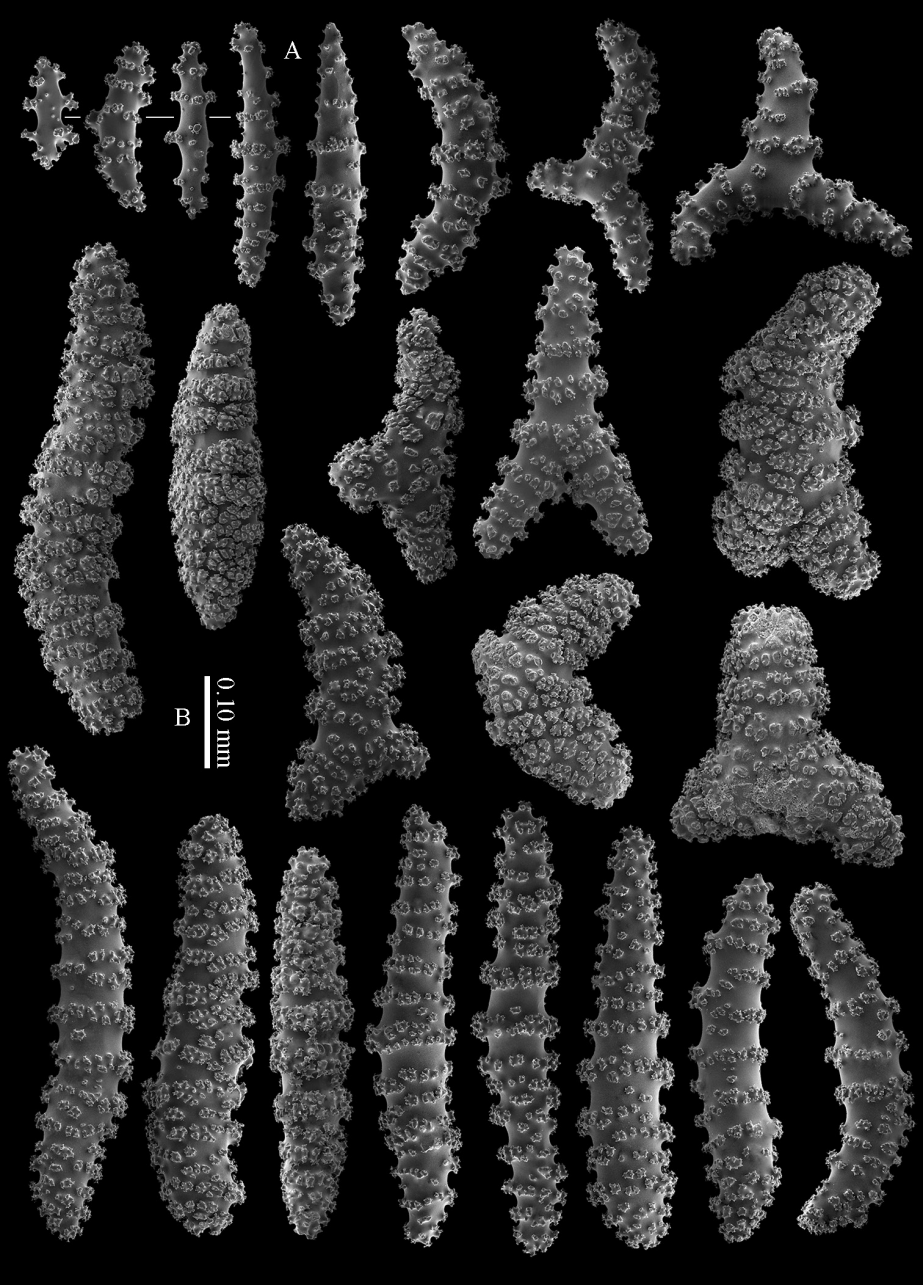
*Briareum cylindrum* sp. n., paratype, RMNH Coel. 41443; **A** sclerites of coenenchyme next to polyp openings **B** cortex sclerites.

**Figure 6.**
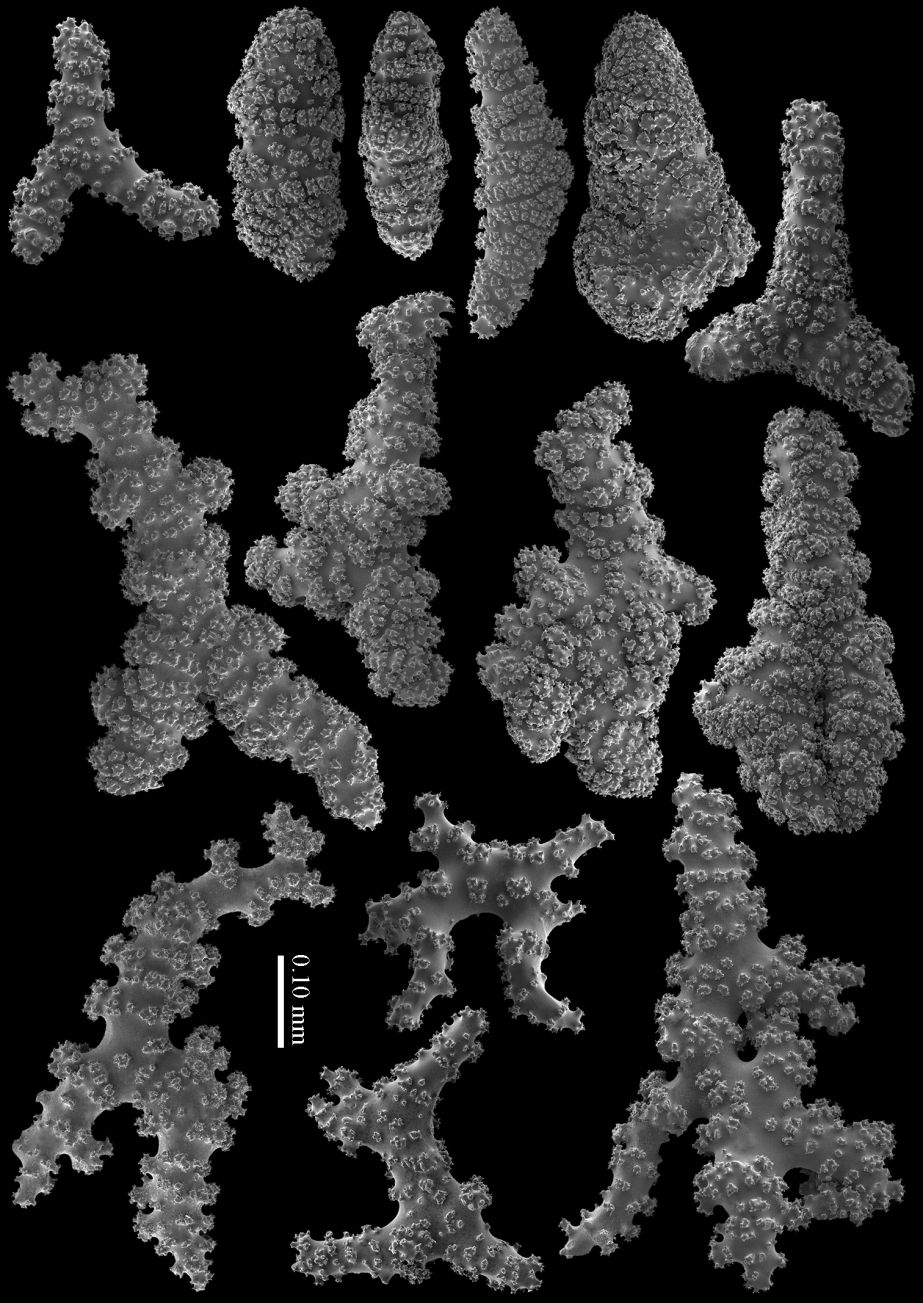
*Briareum cylindrum* sp. n., paratype, RMNH Coel. 41443, medullar sclerites.

**Figure 7.**
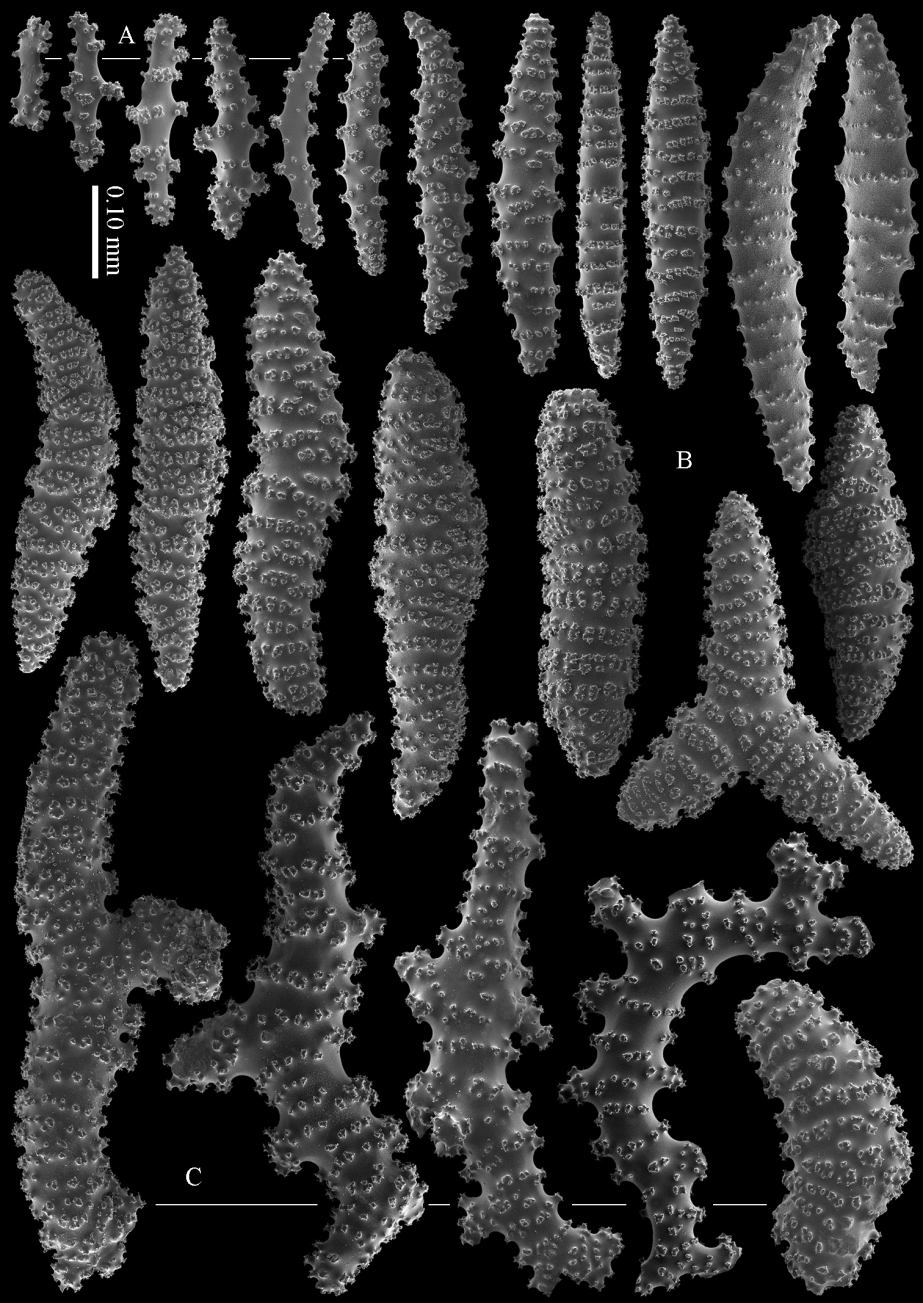
*Briareum cylindrum* sp. n., paratype, RMNH Coel. 32569; **A** sclerites of top calyx **B** cortex sclerites **C** medullar sclerites.

Figures 2A–D , 3 , 4 , 5 , 6 , 7

#### Material examined.

*Holotype*: RMNH Coel. 34193, Malaysia, northwest of channel running due west out of SMART resort, about 100 m away, lobster wall, depth 11 m, 8 July 2004, coll. Nicolas J. Pilcher (0PHG1352–C) (id. *Briareum excavatum*).

*Paratypes*: RMNH Coel. 2241, Indonesia, Java, coll. C.G.C. Reinwardt (id. *Briareum stechei*); RMNH Coel. 2242, 1 microscope slide, Indonesia, Java, coll. C.G.C. Reinwardt, (id. *Briareum stechei*); RMNH Coel. 11655, Australia, Feather Reef, seaward slope, 17°33'S, 146°23'E, depth 0–10 m, 6 July 1975, coll. R.N. Garrett (id. *Briareum stechei*); RMNH Coel. 11797, Australia, Queensland, Great Barrier Reef, Heron Island, on side of Bommie, 15 m depth, 20 July 1973, coll. N. Coleman (id. *Briareum stechei*); RMNH Coel. 13747, Australia, Coral Sea, Mellish Reef, depth 8 m, encrusting on coral block, 1 May 1979, coll. N.L. Bruce, aboard R/V *Lady Basten* (id. *Briareum stechei*); RMNH Coel. 32569, China, Hainan Island, Xidao, 50 km from Haikou City; depth 15 m. October 2003, coll. Wenhan Lin (HSD 9); RMNH Coel. 32570, China, Hainan Island, Xidao, 50 km from Haikou City; depth 15 m. October 2003, coll. Wenhan Lin (HSE 25); RMNH Coel. 41443, Buginesia Progr. UNHAS-NNM 1994/1995, SUL.BCW, Indonesia, southwest Sulawesi, Spermonde Archipelago, west of Barang Caddi (=11 km Northwest of Ujung Pandang = Makassar), 5°05'S, 119°19'E, coral reef, SCUBA diving, 4 May 1994, coll. B.W. Hoeksema; RMNH Coel. 41444, Buginesia Progr. UNHAS-NNM 1994/1995, SUL.KAPN, Indonesia, southwest Sulawesi, Spermonde Archipelago, north of Kapoposang Isl (= 66 km NW of Ujung Pandang = Makassar), 4°40'S, 118°57'E, coral reef, SCUBA diving, coll. B.W. Hoeksema; RMNH Coel. 41446, CEB.05, Philippines, Cebu Strait, west of Bohol, west side of Cabilao Island, south side fish sanctuary, 9°52.60'N 123°45.61'E, dense algae-covered reef flat to 4 m depth, vertical wall with caves to 45 m, SCUBA diving, 8 November 1999, coll. L.P. van Ofwegen; RMNH Coel. 41447, CEB.11, Philippines, Cebu Strait, west of Bohol, east side of Cabilao Island, south of Cambacis, 9°52.92'N 123°47.37'E, to 6 m patchy reef with algae, below steep slope with caves, snorkelling and SCUBA diving, 14 November 1999, coll. L.P. van Ofwegen.

#### Description.

The holotype consists of several fragments of an encrusting colony, the largest being 4 by 1.5 cm in diameter (Figure 2A) with white surface and magenta underside. Calyces hardly projecting.

The calyces contain colourless, flattened rods with prominent simple tubercles (Figure 3A, B). These rods are up to 0.20 mm long. The cortex contains colourless spindles, cylinders, and tripoids (Figure 3C). All these forms have complex tubercles, often arranged in girdles. These sclerites can be up to 0.60 mm long but most are only 0.30 mm long. The medulla contains magenta spindles and branched spindles with simple or complex tubercles (Figure 4). These sclerites are 0.20–0.60 mm long. They can be fused into small clumps.

#### Etymology.

The Latin “cylindrum”, cylinder, refers to the shape of the sclerites.

#### Morphological variation.

RMNH Coel. 13747, RMNH Coel. 32569, RMNH Coel. 41443 and RMNH Coel. 41444 have distinctly longer sclerites with more complex tubercles (Figs 5–7). RMNH Coel. 13747 has slightly raised calyces (Figure 2B); RMNH Coel. 32569 has distinct calyces (Figure 2C), RMNH Coel. 41443 has no calyces at all (Figure 2D).

#### Remarks.

*Briareum cylindrum* mostly resembles *Briareum stechei* but differs in having many cylinders with complex tubercles in the coenenchyme.

#### Distribution.

Australia, Coral Triangle, China. Depth 0–15 m.

### Briareum hamrum

Taxon classificationAnimaliaAlcyonaceaBriareidae

(Gohar, 1948)

**Figure 8.**
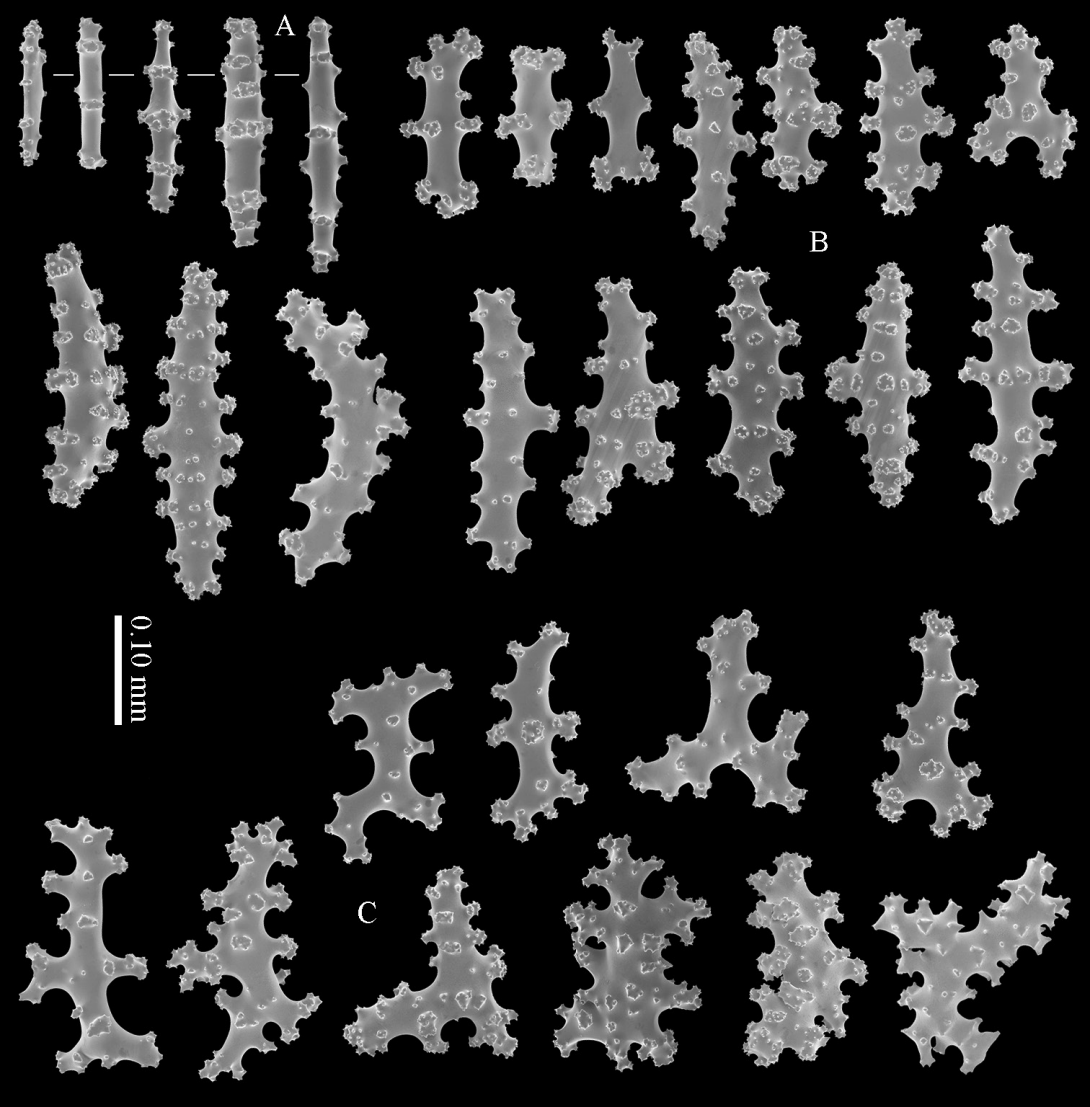
*Briareum hamrum* (Gohar, 1948), RMNH Coel. 6809; **A** sclerites of top calyx **B** cortex sclerites **C** medullar sclerites.

**Figure 9.**
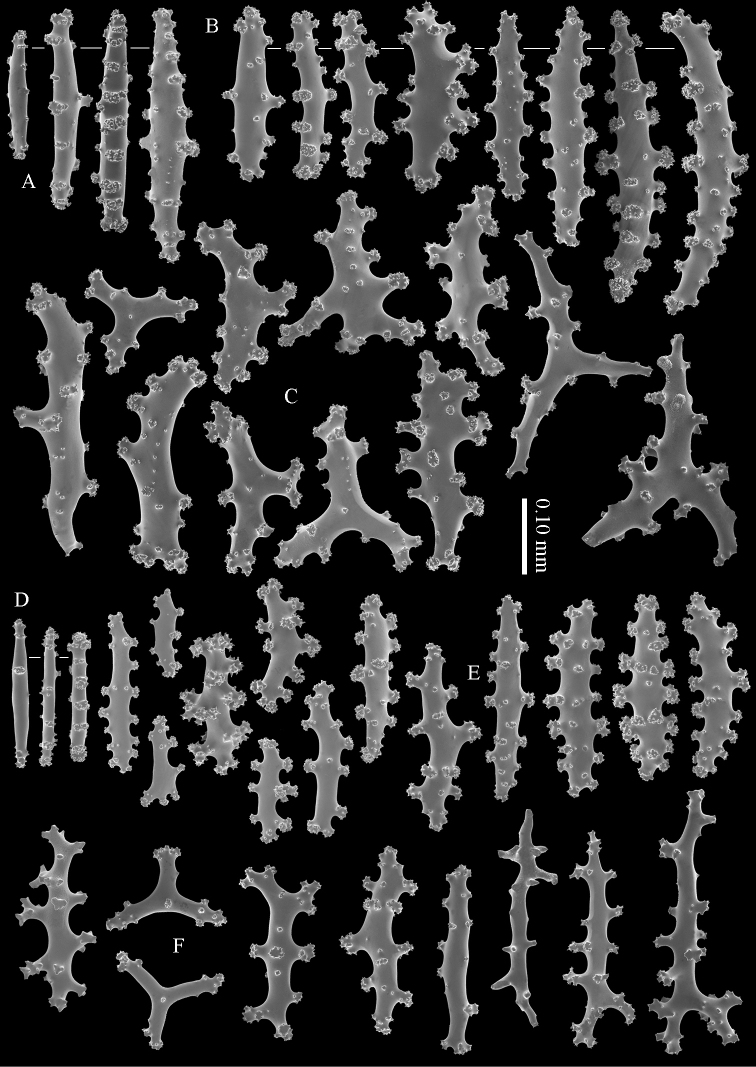
*Briareum hamrum* (Gohar, 1948), RMNH Coel. 41407; **A** sclerites of top calyx **B** cortex sclerites **C** medullar sclerites; RMNH Coel. 41409 **D** sclerites of top calyx **E** cortex sclerites **F** medullar sclerites.

**Figure 10.**
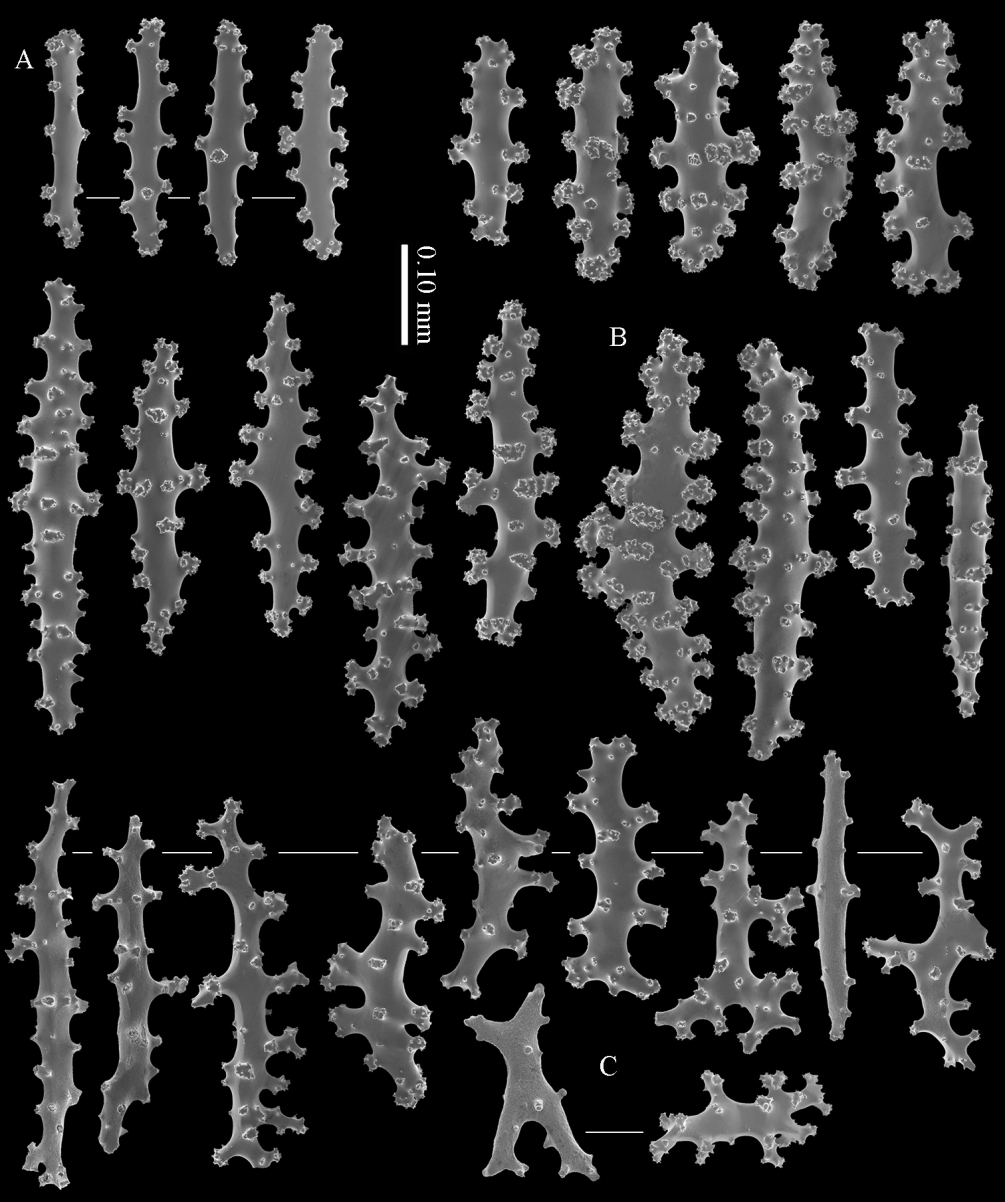
*Briareum hamrum* (Gohar, 1948), RMNH Coel. 41410; **A** sclerites of top calyx **B** cortex sclerites **C** medullar sclerites.

**Figure 11.**
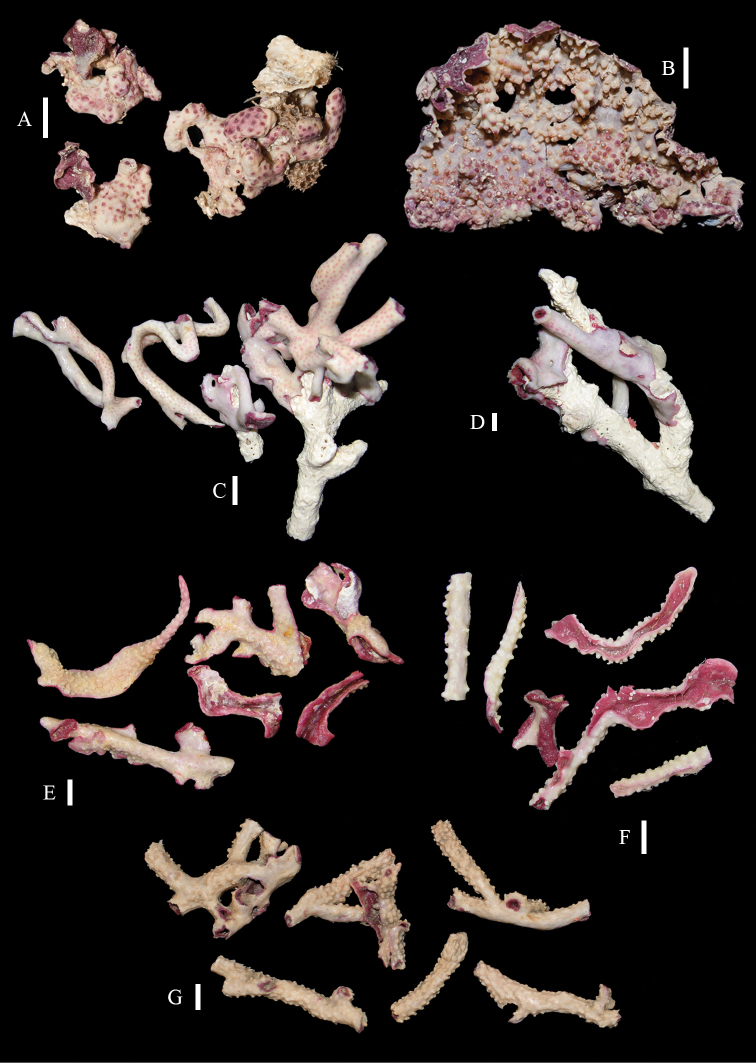
**A–B** Colonies of *Briareum hamrum*; **A** RMNH Coel. 41409 **B** RMNH Coel. 41410 **C–D** *Briareum stechei* **C** ZMB 5828, holotype of *Erythropodium stechei* **D** ZMB 5816 **E–F** Solenopodium stechei var. novaepommeraniae **E** ZMB 5016 **F** ZMB 5854 **G** ZMA 3410, syntype of *Briareum excavatum*. Scale bars: 1 cm.

Figures 8 , 9 , 10 , 2E–F , 11A–B , 26A–B

? Sympodium punctatum May, 1898: 11 (Tumbatu, Zanzibar); Thomson and Henderson 1906: 408, pl. 29 fig. 9 (Chuaka, Tanzania); Tixier-Durivault 1966: 104, figs 96–97 (Madagascar).
? Sympodium splendens Thomson & Henderson, 1906: 409, pl. 29 fig. 8 (Chuaka, Tanzania).
? Alcyonium (Erythropodium) contortum Kükenthal, 1906: 50, pl. 7 figs 34–36, pl. 8 figs 37–38 (Red Sea, Tor, Jimschi).
? Solenopodium contortum Kükenthal, 1919: 41; Stiasny 1937: 10, fig. B (re-examination type).
Clavularia hamra Gohar, 1948: 4, figs 1–5 (Hurghada, Red Sea); Verseveldt 1970: 209 (Eilat).
Solenopodium violaceum Broch & Horridge, 1956: 157 (Hurghada, Red Sea).
Briareum hamrum ; Alderslade 2000: 246; Benayahu et al. 2003: 51 (Bazaruto Island, Mozambique).
Briareum hamra [sic]; Alderslade and McFadden 2007: 42.

#### Material examined.

RMNH Coel. 6809, Red Sea, coll. L.F. Fishelson, NS 6468, det. J. Verseveldt; RMNH Coel. 41406, Madagascar, Tuléar, coll. Nicole Gravier-Bonnet (179), 1967–69, don. H. Zibrowius, Centre d’Oceanologie de Marseille, Station Marine d’Endoume; RMNH Coel. 41407, Iran, Persian Gulf, north of Kish Island, 26°34.512'N 53°59.320'E, 10 m depth, coll. K. Samimi-Namin, 1 October 2009; RMNH Coel. 41408, Iran, Strait of Hormuz, Persian Gulf, north of Larak Island, 26°53.304'N 56°23.769'E, depth 12 m, coll. K. Samimi-Namin, 17 February 2009; RMNH Coel. 41409–41411, Oman, Daymaniyat Islands, 23°51.965'N 58°5.606'E, coll. K. Samimi-Namin; RMNH Coel. 41412, Persian Gulf, north of Farur Island, depth 12–15 m, 10 February 2010, coll. K. Samimi-Namin; RMNH Coel. 41413, Oman, Daymaniyat Islands, 23°51.720'N 58°6.253'E, depth 18 m, coll. K. Samimi-Namin, 23 April 2011; RMNH Coel. 41414, Oman, Daymaniyat Islands, 23°51.720'N 58°6.253'E, depth 18 m, coll. K. Samimi-Namin, 23 April 2011; RMNH Coel. 41415, Oman, Daymaniyat Islands, 23°51.720'N 58°6.253'E, depth 18 m, coll. K. Samimi-Namin, 23 April 2011.

#### Diagnosis.

Calyx with straight spindles containing small tubercles arranged in transverse rows and flattened spindles (Figure 8A). Cortex with straight or bent spindles with complex tubercles (Figure 8B). Coenenchymal sclerites 0.10–0.35 mm long. Medulla additionally has branched sclerites with simple or complex tubercles (Figure 8C). These sclerites are slightly shorter, up to 0.30 mm long. Sclerites of the surface layer are colourless; interior sclerites are magenta.

#### Remarks.

Alderslade (2000) referred *Clavularia hamra* Gohar, 1948 to *Briareum*, consequently the species name had to be changed to *hamrum*.

Gohar (1948: 10) compared his *Clavularia hamra* with both *Sympodium punctatum* May, 1898 and *Sympodium splendens* Thomson & Henderson, 1906, and noticed their close resemblance. According to Gohar (1848), *Sympodium punctatum* differs in having sclerites up to 0.266 mm long while they are up to 0.35 mm long in *Clavularia hamra*. *Sympodium splendens* differs in having two rows of pinnules on either side of the tentacles, each row consisting of 20–24 pinnules, while in *Clavularia hamra* there is only one row of 16–22 pinnules, which are much longer. However, an odd second row of 1–3 pinnules can be present in *Clavularia hamra*. Furthermore, *Clavularia hamra* has no triradiate or tetraradiate sclerites, described for *Sympodium splendens*. Next to the radiates Thomson and Henderson (1906) described the sclerites to be straight and curved spindles, up to 0.4 mm long. From our material and findings of Prof. Y. Benayahu (see Alderslade 2000: 246) it seems only one *Briareum* species is present in the Red Sea and the western Indian Ocean. Consequently, the correct name should be the oldest available, *Briareum punctatum* May, 1898, but the type material of *Briareum punctatum* is missing. As we had no material from its type locality, Zanzibar, we could not designate a proper neotype yet. As the species was never again found in Zanzibar we still have some doubts about its identity and thus defer to *Briareum hamrum* for the moment. Notably, also the type material of *Sympodium splendens*, Alcyonium (Erythropodium) contortum and *Briareum hamrum* seems to be missing.

This is the first record of a *Briareum* species from the Persian Gulf, and Oman Sea (see Samimi-Namin and van Ofwegen 2009, 2012).

#### Morphological variation.

RMNH Coel. 41407 (Figure 2F) from the Persian Gulf differs from the above described Red Sea specimen. It has longer sclerites (up to 0.40 mm long; Figure 9B) and more slender interior branched bodies (Figure 9C). RMNH Coel. 41410 (Figure 11A) from Oman has even longer sclerites than the Persian Gulf specimen (up to 0.45 mm long; Figure 10); it is the only specimen having long calyces. RMNH Coel. 41409 (Figure 11B), also from Oman, has sclerites (Figure 9D–F) with the same size as the Red Sea specimen, but the slender interior branched bodies as the Persian Gulf and other Oman specimen. RMNH Coel. 41412 has completely colourless sclerites, however, the colour of live specimens was similar to others. The shape of the colonies in the examined material showed variation, from completely encrusting to somewhat having branches and an undulated surface.

#### Colour.

The living colonies were cream with magenta tints in some parts of the colony. Polyps were dark green to brown, brown pinnules, white oral disk and white line that continues along the tentacles (Figure 26A–B)

#### Distribution.

Red Sea, East Africa, Oman Sea, Arabian Sea, Persian Gulf.

### Briareum stechei

Taxon classificationAnimaliaAlcyonaceaBriareidae

(Kükenthal, 1908)

Figures 11C–G , 12 , 13 , 14 , 15 , 16 , 17 , 18 , 26C–D

Erythropodium stechei Kükenthal, 1908: 19 (Banda); 1919: 38.
Suberia excavata Nutting, 1911: 14, pl. 3 fig. 2, 2a, pl. 11 fig. 4 (Ambon).
Solenopodium excavatum ; Kükenthal 1919: 42; 1924: 13; Stiasny 1937: 12, Pl. 1 figs 4–5, fig. C (re-examination type); Verseveldt 1940: 32–37.
Solenopodium stechei var. novaepommeraniae Kükenthal, 1919: 901 (New Britain); 1924: 13.
Solenopodium stechei ; Aurivillius 1931: 9 (Timor); Stiasny 1937: 17, pl. 1 figs 1–3, fig. E (re-examination syntype ZMB 5828); Macfadyen 1936: 67 (Australia); Tixier-Durivault 1970: 325 (New Caledonia).
Briareum excavatum ; Benayahu 1997: 238 (Guam); Erhardt and Baensch 2000: 220 (life image, RMNH Coel. 24018); Benayahu et al. 2004: 551, fig. 3 (Taiwan).
Briareum stechei ; Grasshoff 1999: 6, fig. 5 (New Caledonia).
Briaeum [sic] *excavatum*Benayahu 2002: 20 (Ryukyu Archipelago, Japan).
Briareum cf. stechei ; Alderslade and McFadden 2007: 41, fig. 10B.

#### Material examined.

ZMB 5828, holotype *Erythropodium stechei*: Banda Island (Moluccas), litoral, leg. Steche; ZMB 5816, Ambon (Moluccas), litoral, leg. Steche; ZMB 5016, 5854, holotype Solenopodium stechei var. novaepommeraniae, Neupommern, litoral, leg. Schoede; ZMA 3410, syntype *Briareum excavatum*; Siboga Exped. stat. 142, Maluku, anchorage off Laiwui, depth 23 m, Hensen vertical net, tow net, dredge; ZMA 3489, same data as ZMA 3410; RMNH Coel. 5837, Indonesia, Laiwui, Obi Major St. 142 Siboga expedition (id. *Solenopodium excavatum*); RMNH Coel. 18416, Indonesia, Celebes, Westside Samalona, 18 m depth, 18 September 1980, coll. H. Moll (id. *Briareum excavatum*); RMNH Coel. 41416, Indonesian-Dutch Snellius II Exp., Sta. 4.052, NE coast of Sumba, E of Melolo, 09°55'S, 120°45'E, edge of extensive, gently sloping reef flat, SCUBA diving, snorkelling, depth 10–15 m, 13/14 September 1984; RMNH Coel. 41417, Indonesian-Dutch Snellius II Exp., Sta. 4.222, northeast Taka Bone Rate (tiger islands), south of Pulau Tarupa Kecil, rectangular dredge, depth 58 m, 06°31.5'S, 121°08.0'E, sandy bottom with gorgonians antipatharians, sponges, 14 October 1984; RMNH Coel. 41418, Buginesia Progr. UNHAS-NNM 1994/1995, Sta. SUL.SAM S, Indonesia, southwest Sulawesi, Spermonde Archipelago, south of Samalona Isl. (= 7.5 km W of Ujung Pandang = Makassar), 5°07'S, 119°20'E; coral reef; SCUBA diving, 31 May 1994, coll. B.W. Hoeksema; RMNH Coel. 41419, BUN.06, Indonesia, North Sulawesi, Tanjung Totrowitan main coast, steep slope fringing reef, 01°45'N 124°58.500'E, SCUBA diving. 6 May 1998, coll. B.W. Hoeksema and L.P. van Ofwegen; RMNH Coel. 24018, Indonesia, Banda Island, depth 12 m, 30 September 1998, coll. H. Erhardt, dry material, det. L.P. van Ofwegen (id. *Briareum excavatum*); RMNH Coel. 41420–21, RUM.20, Indonesia, Moluccas, Ambon, Hitu, north coast, Hitulama; 20 November 1990, coll. C.J.H.M Fransen; RMNH Coel. 41422, BUN.14, Sta.14: Indonesia, N. Sulawesi, Bunaken Park, NE Bunaken Island, steep slope,124°46'30”E 01°36'30”N, SCUBA diving. 10. May 1998; Coll. B.W. Hoeksema; RMNH Coel. 41423, CEB.08, Philippines, Cebu Strait, west of Bohol, north side of Cabilao Island, Cabacungan Point, 9°51.55'N 123°45.95'E, reef edge with dense coral cover, overhanging wall with caves, snorkelling and SCUBA diving, 11 November 1999, coll. L.P. van Ofwegen; RMNH Coel. 41424, BER.16, Indonesia, northeast Kalimantan, Berau Islands, Maratua Island, NE-side, 2°17.487'N 118°35.483'E, SCUBA diving, 10 October 2003, depth 28 m, coll. L.P. van Ofwegen and M. Slierings; RMNH Coel. 38607, Sabah, Layang Layang atoll, outer reef on east end of atoll, 07°22.69'N 113°52.23'E, depth 10 m, 13 October 2006 (0CDN 9322–R); RMNH Coel. 39998, Palau, Angaur, northeast side of island, reef sloping 30° to sandy slope below 200 ft., reef top, rock, depth 8 m, 06°55.36'N 134°08.68'E, 21 June 2008 (0CDN9600–T); RMNH Coel. 40023; Palau, SW Islands, Helen Reef lagoon, reef of conservation area main lagoon marker, lagoon patch reef, large with shallower reef area, sand/rubble/coral patches, 16 September 2008, depth 14 m, lagoon Pinnacle, Rock, 02°52.860'N 131°46.510'E (0CDN9778–N); RMNH Coel. 40078, Palau, Velasco Reef north of Kayangel, central ‘lagoon’ area; pinnacle/large patch reef 15 m depth in mid ‘lagoon’ of Velasco reef Lagoon pinnacle, rock depth 15 m, 08°17.290'N 134°38.200'E; 26 June 2009 (0CDN9988–Q); RMNH Coel. 41425, Exp. Indonesia, Ternate – Halmahera. 2009, TER.15, Indonesia, Halmahera, Tidore, Cobo, 0°45.312'N 127°24.397'E, 01 November 2009; RMNH Coel. 40884, PAL.168, Republic of Palau, Koror, Wonder Channel, 7°10.869'N 134°21.612'E, depth 19.6 m, 21 May 2010, coll. C.S. McFadden; RMNH Coel. 40885, PAL.173, Republic of Palau, Koror, Wonder Channel, 7°10.869'N, 134°21.612'E, depth 18.8 m, 21 May 2010, coll. C.S. McFadden; RMNH Coel. 40886, PAL.218, Republic of Palau, Koror, Pinchers, 7°20.402'N 134°25.682'E, depth 7.3 m, 22 May 2010, coll. C.S. McFadden; RMNH Coel. 40887, PAL.314, Republic of Palau, Koror, Turtle Cove, 7°05.078'N 134°15.730'E, depth 52 m, 24 May 2010, coll. C.S. McFadden; RMNH Coel. 41445, CEB.05, Philippines, Cebu Strait, W of Bohol, west side of Cabilao Island, south side fish sanctuary, 9°52.60'N 123°45.61'E, dense algae-covered reef flat to 4 m, vertical wall with caves to 45 m, SCUBA diving, 8 November 1999, coll. L.P. van Ofwegen; RMNH Coel. 41448, BER.03, Indonesia, northeast Kalimantan, Berau Islands, Derawan Island, southern side (jetty Derawan Dive Resort), 2°17.055'N 118°14.813'E, SCUBA diving, 22 October 2003, depth 20 m, coll. L.P. van Ofwegen and M. Slierings; RMNH Coel. 41615, TER.32, Indonesia, Pulau Pulau Gura Ici, east Pulau Gura Ici, 0°1.288'S, 127°14.287'E, 10 November 2009, depth 18 m, coll. B.T. Reijnen.

#### Diagnosis.

**Figure 12.**
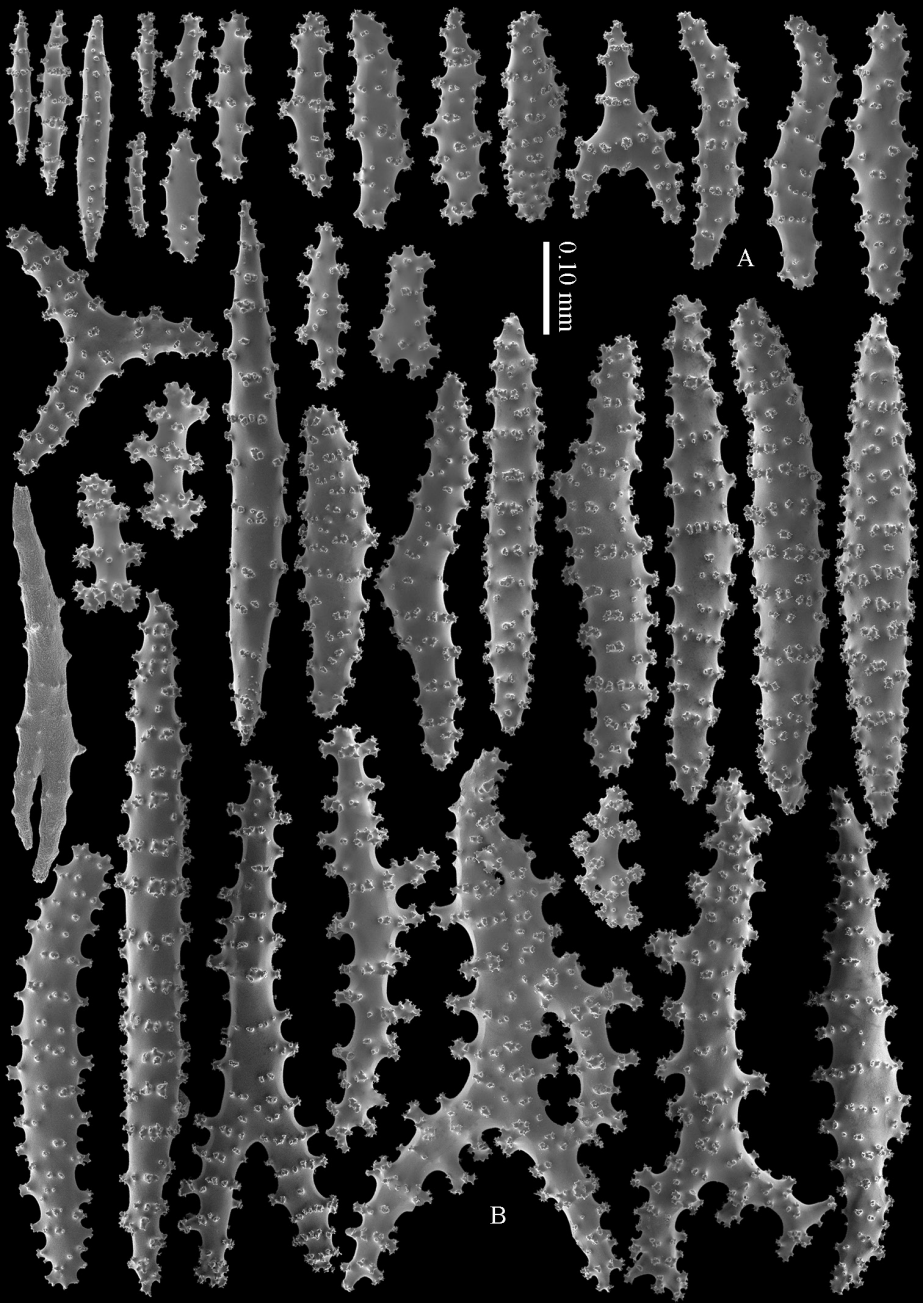
*Briareum stechei* (Kükenthal, 1908), ZMB 5828, holotype; **A** cortex sclerites **B** medullar sclerites.

Cortex with straight or bent spindles with simple or complex tubercles mostly arranged in transverse rows (Figure 12A). These cortex sclerites are 0.10–0.75 mm long. The medulla additionally has branched bodies with simple or complex tubercles (Figure 12B). These sclerites are slightly shorter, up to 0.60 mm long. Sclerites of surface layer colourless, interior sclerites magenta.

#### Remarks.

The sclerites are most like those of *Briareum violaceum* but in that species many spindles are longer than the longest of *Briareum stechei*.

**Figure 13.**
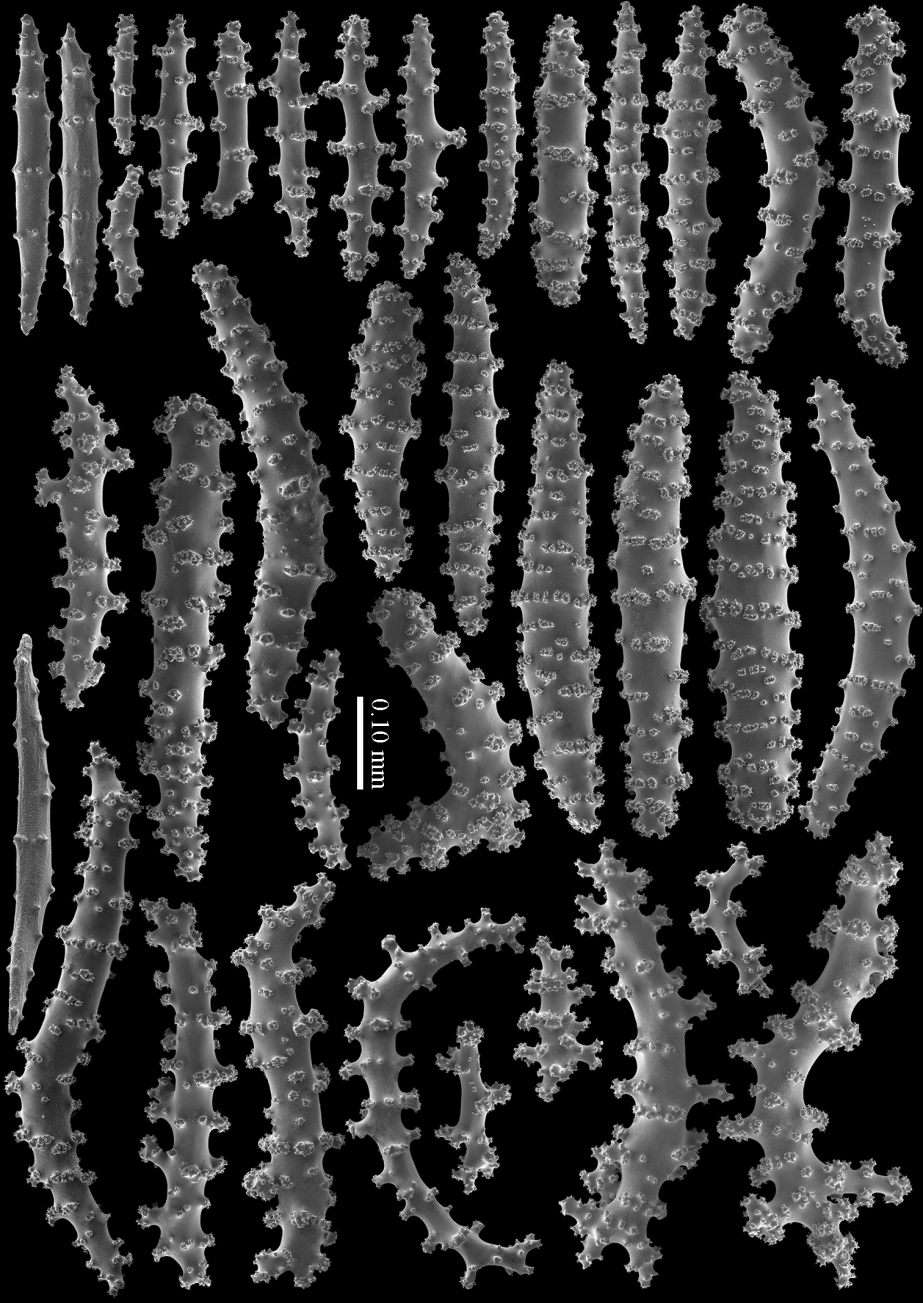
*Briareum stechei* (Kükenthal, 1908), ZMA 3410, syntype of *Briareum excavatum*; **A** cortex sclerites **B** medullar sclerites.

Nutting (1911) apparently was not aware of Kükenthal’s earlier (1908) description of *Erythropodium stechei*; actually, at first he did not compare his new species with any previously described one. Later, Kükenthal (1919) noticed the resemblance with his *Erythropodium stechei*, now in the genus *Solenopodium* Kükenthal, 1916a, and put it in the synonymy of that species with a question mark as he did not re-examine Nutting’s material. Stiasny (1937) re-examined type material of both *Solenopodium stechei* and *Solenopodium excavatum* and kept them as separate species. According to him, *Solenopodium excavatum* differs in having higher calyces (Figure 11G), and by lacking calyx sclerites and “dendritic” sclerites in the interior. Verseveldt (1940: 37) was the last to compare these two species and noted no less than six aspects of difference between them, however, he did not re-examine the type material of *Briareum stechei*. We present sclerites images of the types of the two species (Figures 12–13). We consider the differences mentioned by previous authors as intraspecific variation, similar to that as observed in *Briareum hamrum*, and therefore we synonymize *Briareum excavatum* with *Briareum stechei*.

Kükenthal (1908) described *Erythropodium stechei* from Banda only. In the Berlin Museum, ZMB 5828 (Fig. 11C), material from Banda, and ZMB 5816 (Figure 11D), material from Ambon, are present, both labelled type. It looks like these specimens represent the same material. It is puzzling to us why Ambon is now mentioned as the locality of ZMB 5816.

Solenopodium stechei var. novaepommeraniae is also represented by two collection numbers in Berlin, ZMB 5016 (Figure 11E) and ZMB 5854 (Figure 11F), here obviously the original material was split into two.

#### Morphological variation.

**Figure 14.**
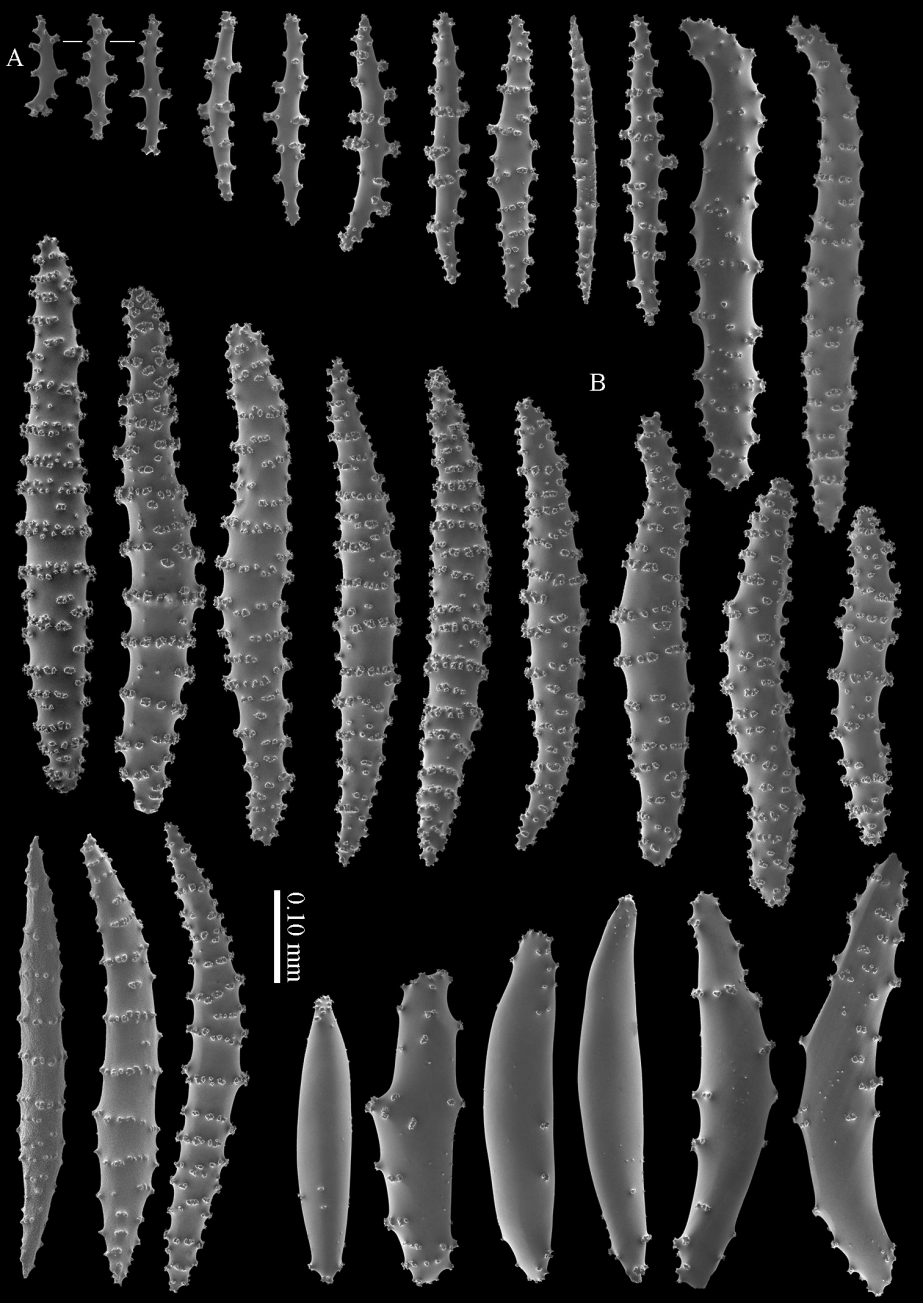
*Briareum stechei* (Kükenthal, 1908), RMNH Coel. 40023; **A** sclerites of top calyx **B** cortex sclerites.

**Figure 15.**
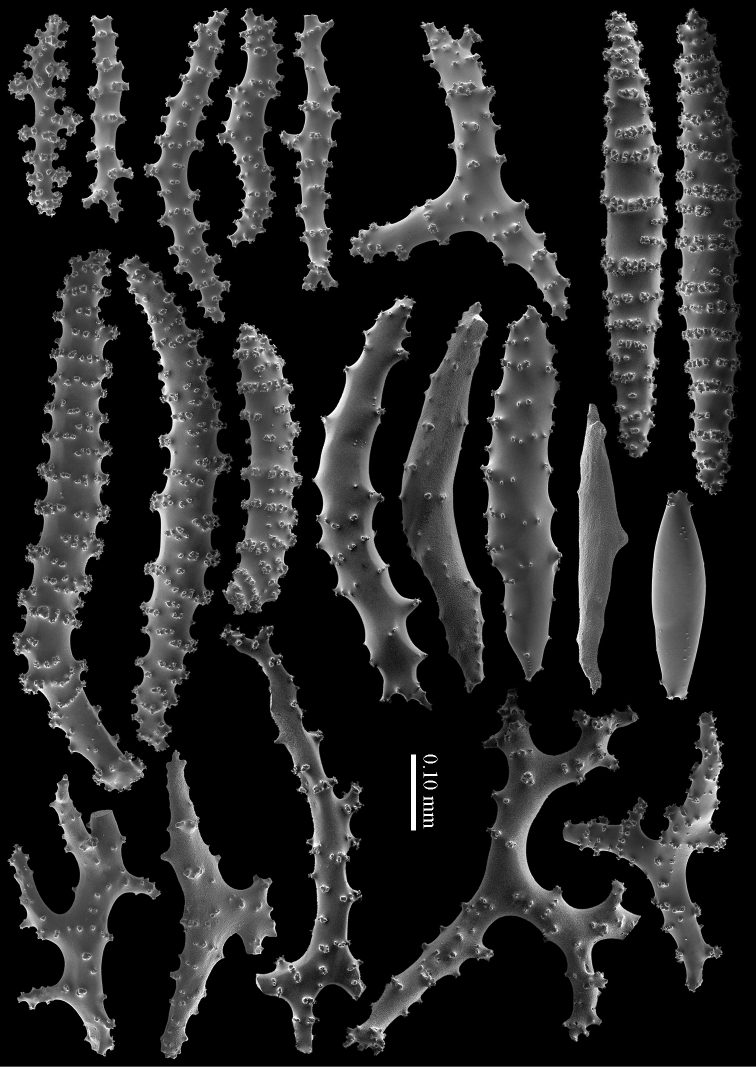
*Briareum stechei* (Kükenthal, 1908), RMNH Coel. 40023; medullar sclerites.

**Figure 16.**
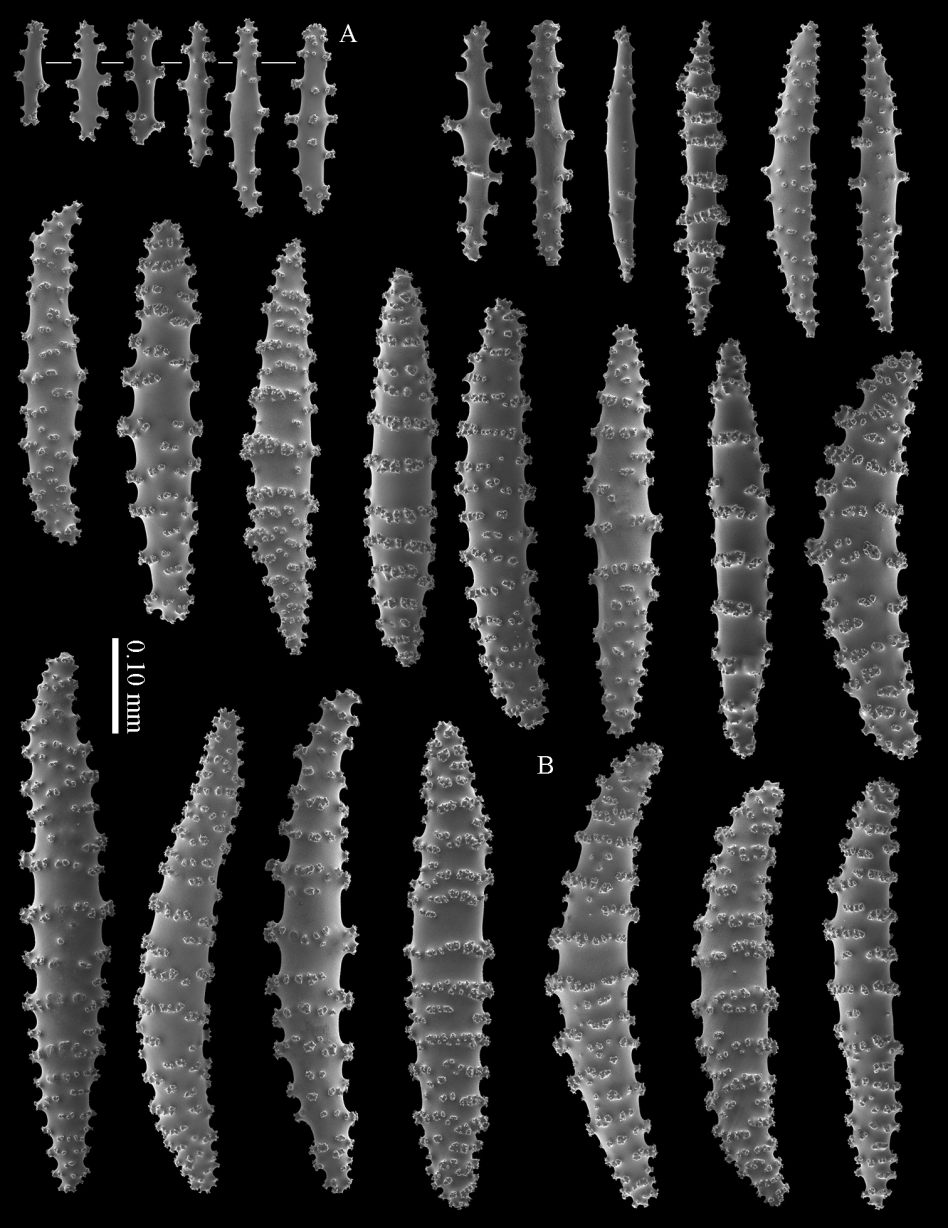
*Briareum stechei* (Kükenthal, 1908), RMNH Coel. 40023; **A** sclerites of top calyx **B** cortex sclerites.

**Figure 17.**
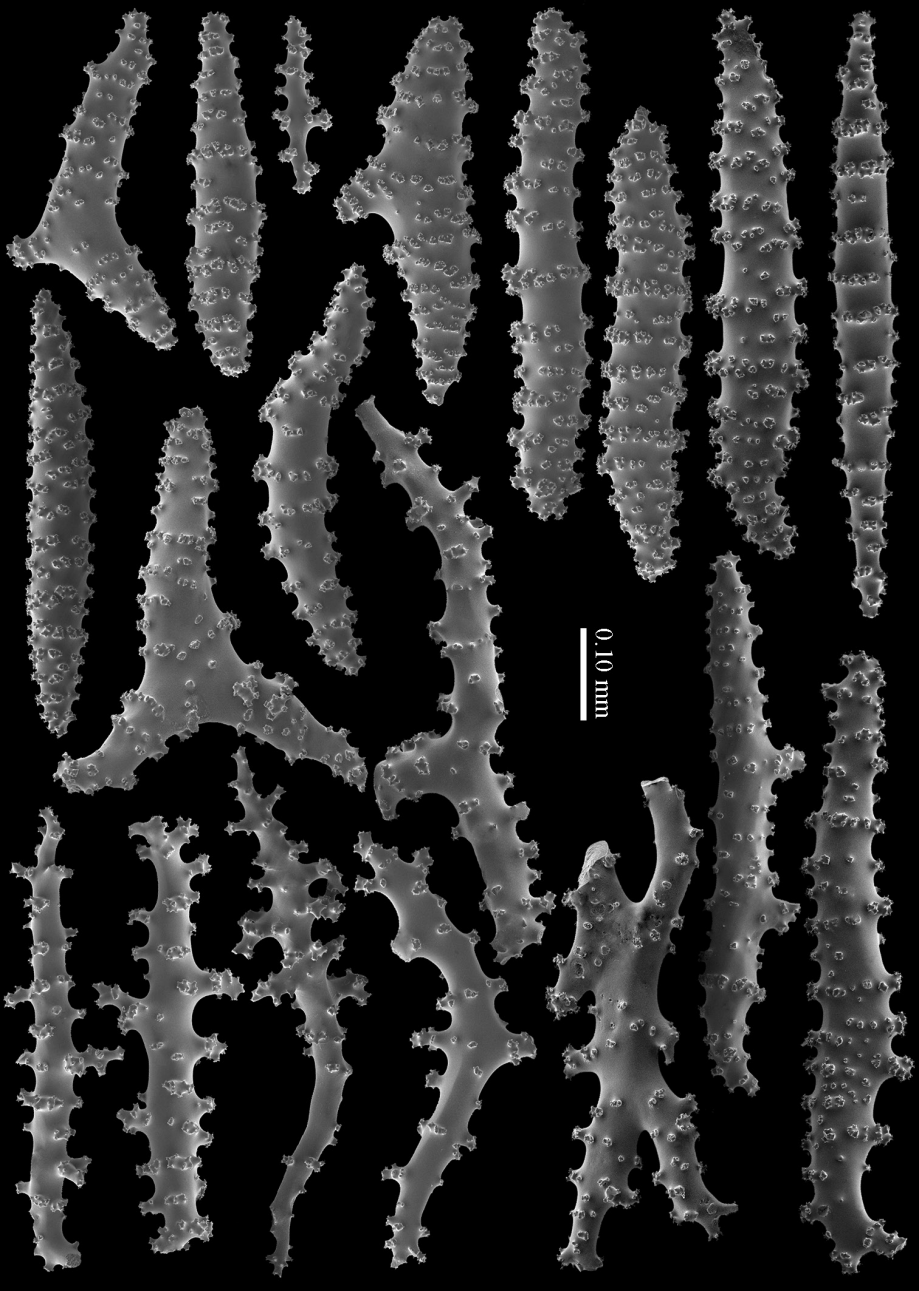
*Briareum stechei* (Kükenthal, 1908), RMNH Coel. 40023; medullar sclerites.

**Figure 18.**
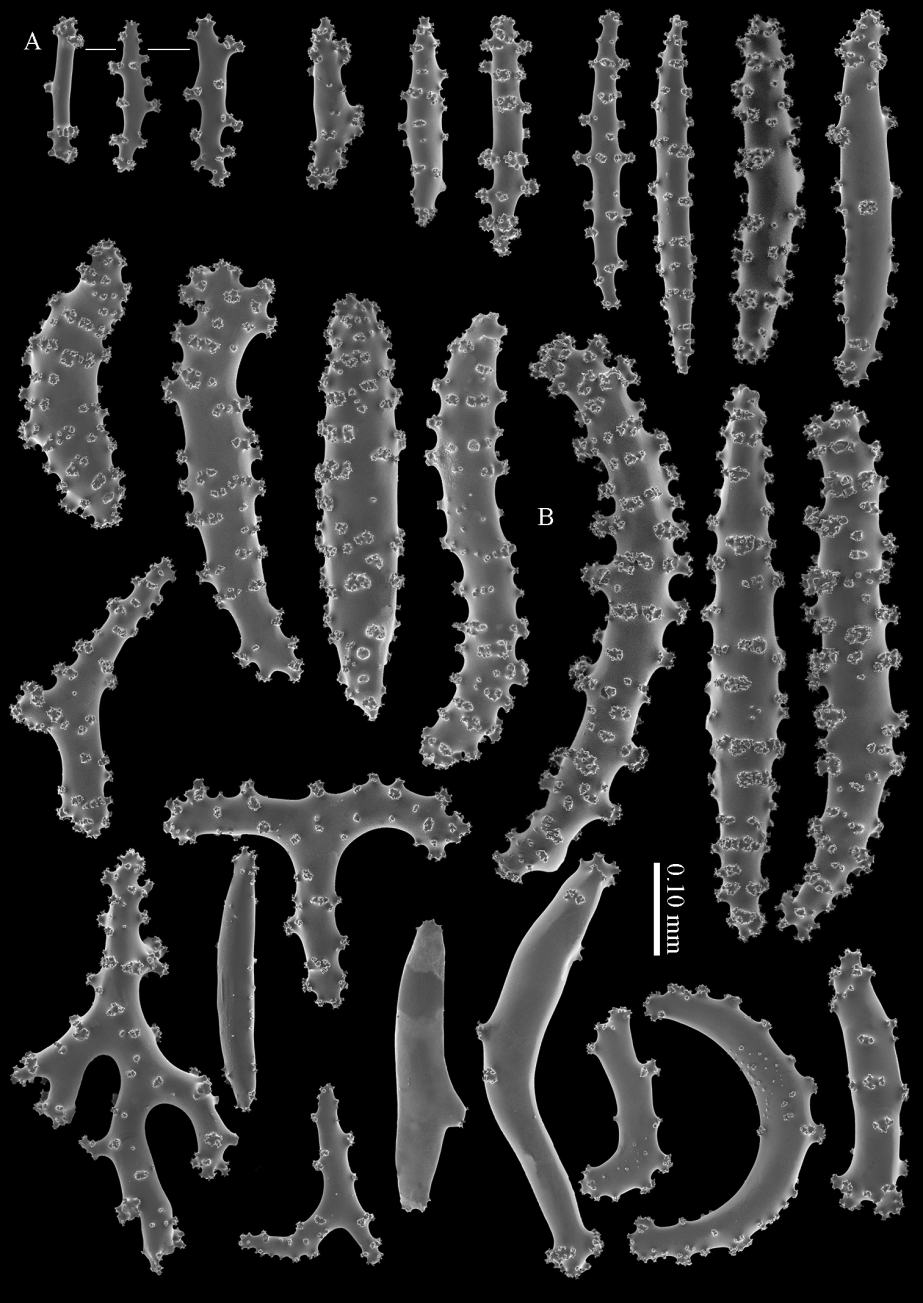
*Briareum stechei* (Kükenthal, 1908), RMNH 41421; **A** sclerites of top calyx **B** sclerites of coenenchyme.

To show the enormous variation in sclerites we have made SEM images of two specimens from Palau (RMNH Coel. 40023) collected at the same locality. One of them shows almost smooth spindles (Figs 14–15) while the other, like the type, has none at all (Figs 16–17). RMNH Coel. 41421 has peculiar bent and smooth sclerites (Figure 18).

#### Distribution.

Coral Triangle, Australia (Low Isles), Guam, Taiwan.

### Briareum violaceum

Taxon classificationAnimaliaAlcyonaceaBriareidae

(Quoy & Gaimard, 1833)

Figures 19 , 20 , 21 , 22 , 23 , 24 , 25 , 26E–F

Clavularia violacea Quoy & Gaimard, 1833: 262, pl. 21 figs 13–16 (Solomon Islands).
Pachyclavularia erecta Roule, 1908: 165, pl. 6 figs 4–5 (Ambon); Thomson and Dean 1931: 19, pl. 2 figs 4, 8–9, pl. 5 figs 6–7, 9, pl. 16 figs 1–2 (Indonesia); Macfadyen 1936: 20 (Great Barrier Reef); Imahara 1996: 19 (Japan).
Pachyclavularia violacea ; Gohar 1940: 20; Utinomi 1956: 223 (Bonin Islands), 1959 (Taiwan); Verseveldt 1960: 211 (Indonesia), 1972: 457 (Marshall Islands); Utinomi 1976: 3; Benayahu 1995: 106 (Sesoko Island, Japan); van Ofwegen 1996: 207 (Bismarck Sea); Imahara 1996: 19 (Okinawa, Japan).
Briareum violacea ; Benayahu 2002: 20 (Ryukyu Archipelago, Japan); Benayahu et al. 2004 (South Taiwan).
Briareum violaceum ; Alderslade and McFadden 2007: 42.
Not Solenopodium violaceum Broch & Horridge, 1956: 157 (= *Briareum hamrum*; Red Sea).

#### Material examined.

RMNH Coel. 38608, Sabah, Layang Layang Atoll, outer reef on east-end of atoll, 07°22.69'N 113°52.23'E, depth 5 m, 13 October 2006 (0CDN 9323–S); RMNH Coel. 40883, PAL.100, 21 May 2010 Palau, Koror, Siaies Tunnel, 7°18.686'N,134°13.596'E, 31 m depth, coll. C.S. McFadden; RMNH Coel. 40001, Palau, Northern Reefs, northwest corner, just east of reef tip, slope, rock, 29 June 2008, depth 20 m, 07°58.96'N, 134°34.39'E (0CDN9611–H); RMNH Coel. 41426, MAL.04 Indonesia, Ambon, Outer bay, south coast northeast of Cape Hahurong, 03°47'S, 128°06'E, calcareous platforms in littoral and shallow sublittoral, rather steep slope with more than 50% coral cover; snorkelling and diving, depth 10–27 m; 6 November 1996, coll. L.P. van Ofwegen; RMNH Coel. 41427, BUN.15, Indonesia, N. Sulawesi, Bunaken Park (main coast), Tanjung Pisok, reef flat, 124°48'E 01°34'N, SCUBA diving. 11 May 1998, coll. B.W. Hoeksema and L.P. van Ofwegen; RMNH Coel. 41428, CEB.08, Philippines, Cebu Strait, west of Bohol, north side of Cabilao Island, Cabacungan Point, 9°51.55'N 123°45.95'E, reef edge with dense coral cover, overhanging wall with caves; snorkelling and SCUBA diving, 11 November 1999, coll. L.P. van Ofwegen; RMNH Coel. 41429, CEB.09, Philippines, Cebu Strait, W of Bohol, north side of Cabilao Island, NE of Looc, 9°53.59'N 123°46.92'E, reef edge with dense coral cover, overhanging wall with caves; snorkelling and SCUBA diving, 12, 13 November 1999, coll. L.P. van Ofwegen;. RMNH Coel. 41430, Bali Lombok Strait Exp. 2001, NNM-LIPI-WWF, BAL.04, Indonesia, Bali, Sanur, Jeladi Willis, south of channel entrance; 8°40.983'S, 115°16.050'E, slowly declining shallow reef slope, sandy base; SCUBA-diving to 10 m depth; 1 April 2001, coll. L.P. van Ofwegen and M. Slierings; RMNH Coel. 41431, Indonesia Ambon. Bali Lombok Strait Exp. 2001, NNM-LIPI-WWF, BAL.06, Indonesia, Bali, Sanur, Bangsal Point; 8°40.233'S, 115°15.867'E, slowly declining shallow reef slope, sandy base; SCUBA diving to 9 m depth; 2 April 2001, coll. L.P. van Ofwegen and M. Slierings; RMNH Coel. 41432, Kepulauan Seribu Exped. 2005, SER.23, Indonesia, Java Sea, Kepulauan Seribu (Thousand Islands), off Jakarta, Jukung Island, northwest side, 5°34.017'S, 106°31.633'E, SCUBA diving and snorkelling, 15 September 2005, coll. L.P. van Ofwegen and M. Slierings; RMNH Coel. 41433, Kepulauan Seribu Exped. 2005, SER.25, Indonesia, Java Sea, Kepulauan Seribu (Thousand Islands), off Jakarta, Kotok Kecil Island, northwest side, 5°41.933'S, 106°32.383'E, SCUBA diving and snorkelling, 16 September 2005; RMNH Coel. 41434, Buginesia Progr. UNHAS-NNM 1994/1995, SUL.BTN, Indonesia, SW Sulawesi, Spermonde Archipelago, north of Bone Tambung (= 17 km NW of Ujung Pandang = Makassar), 5°02'S, 119°16'E, coral reef; SCUBA diving, 14 May 1994, coll. B.W. Hoeksema; RMNH Coel. 41435, CEB.13, Philippines, Cebu Strait, W of Bohol, N side of Sandingan Island, 9°51.87'N 123°47.76'E, 0–7 m sandy, patchy coral cover, 7–24 m rubble slope, snorkelling and SCUBA diving; 13 November 1999, coll. L.P. van Ofwegen; RMNH Coel. 41436, Kepulauan Seribu Exped. 2005, SER.06, Indonesia, Java Sea, Kepulauan Seribu (Thousand Islands), off Jakarta, Dapur Island, northwest side, 5°56.733'S, 106°43.450'E, SCUBA diving and snorkelling, 9 September 2005; RMNH Coel. 41437, Indonesia Ambon. Bali Lombok Strait Exp. 2001, NNM-LIPI-WWF, BAL.25, Indonesia, Bali, northwest of Nusa Lembongan, Lembongan Bay, Bali Hai pontoon, off Desa Jungutbatu, 8°40.417'S, 115°26.300'E; shallow bay with few patches of coral; SCUBA-diving to 12 m depth; 17 April 2001, coll. L.P. van Ofwegen and M. Slierings; RMNH Coel. 41438, MAL.04 Indonesia, Ambon, Outer bay, south coast northeast of Cape Hahurong, 03°47'S, 128°06'E; calcareous platforms in littoral and shallow sublittoral, rather steep slope with more than 50% coral cover; snorkelling and diving, depth 2–28 m, 6 November 1996, coll. L.P. van Ofwegen; RMNH Coel. 41439, RAJ.12, Indonesia, Raja Ampat Islands, W Papua, east Kri, Sorido Wall, 00°33.220'S, 130°41.282'E, 22 November 2007, depth 30 m, coll. L.P. van Ofwegen; RMNH Coel. 41440, BUN.08, Indonesia, north Sulawesi, Bunaken Park, south Manado Tua Island, vertical wall in front of church, 1°37.000'N 124°41.500'E, SCUBA diving 15 m, 7 May 1998, coll. B.W. Hoeksema and L.P. van Ofwegen; RMNH Coel. 41441, BER.15, Indonesia, northeast Kalimantan, Berau Islands, Panjang Island, west side, 2°19.285'N 118°13.425'E, SCUBA diving, 9 October 2003, depth 20 m, coll. L.P. van Ofwegen and M. Slierings; RMNH Coel. 41442, Bali Lombok Strait Exp. 2001, NNM-LIPI-WWF, BAL.12, Indonesia, Bali, east side Nusa Dua, off Club Med Hotel, north of channel, 8°47.100'S, 115°13.950'E; slowly declining reef slope, sandy base; SCUBA diving to 20 m depth; 4 April 2001, coll. L.P. van Ofwegen and M. Slierings.

#### Diagnosis.

Top of the calyces with some rods, 0.10–0.15 mm long (Figure 20A). Cortex with straight and bent spindles with simple or complex tubercles arranged in rows (Figure 20B). Cortex sclerites up to 1 mm long. Interior additionally has branched bodies with simple or complex tubercles (Figure 20C), some fused into small clumps. These sclerites slightly shorter, up to 0.70 mm long. All sclerites magenta.

#### Remarks.

Gohar (1940: 20) synonymized *Pachyclavularia erecta* with *Pachyclavularia violacea*. The type of *Briareum violaceum* is stored in Paris. It was not re-examined by us.

#### Morphological variation.

**Figure 19.**
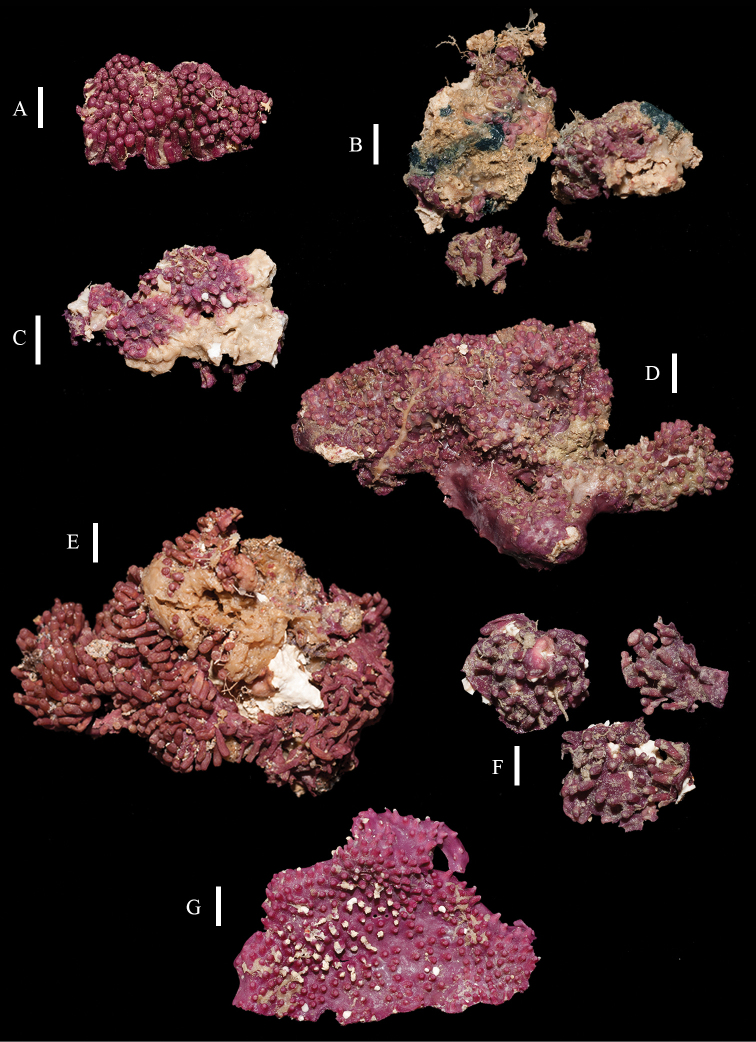
Colonies of *Briareum violaceum*; **A** RMNH Coel. 38608 **B** RMNH Coel. 41435 **C** RMNH Coel. 41434 **D** RMNH Coel. 41436 **E** RMNH Coel. 41437 **F** RMNH Coel. 41441 **G** RMNH Coel. 41428. Scale bars: 1 cm.

RMNH Coel. 41435 (Figure 19B) showed sclerites with very small and simple tubercles (Figure 21).

**Figure 20.**
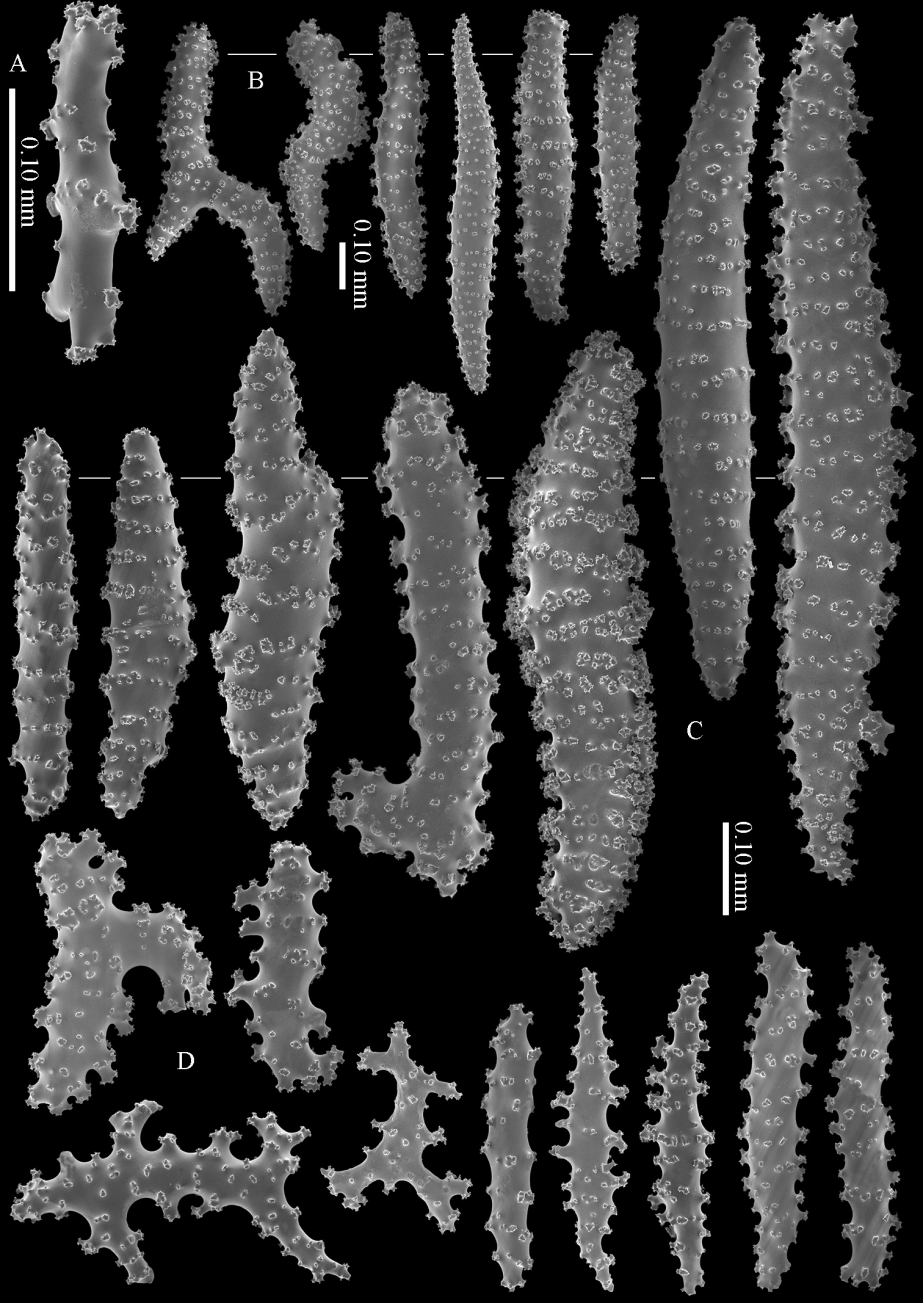
*Briareum violaceum* (Quoy & Gaimard, 1833), RMNH 38608; **A** sclerite of top calyx **B–C** cortex sclerites **D** medullar sclerites.

**Figure 21.**
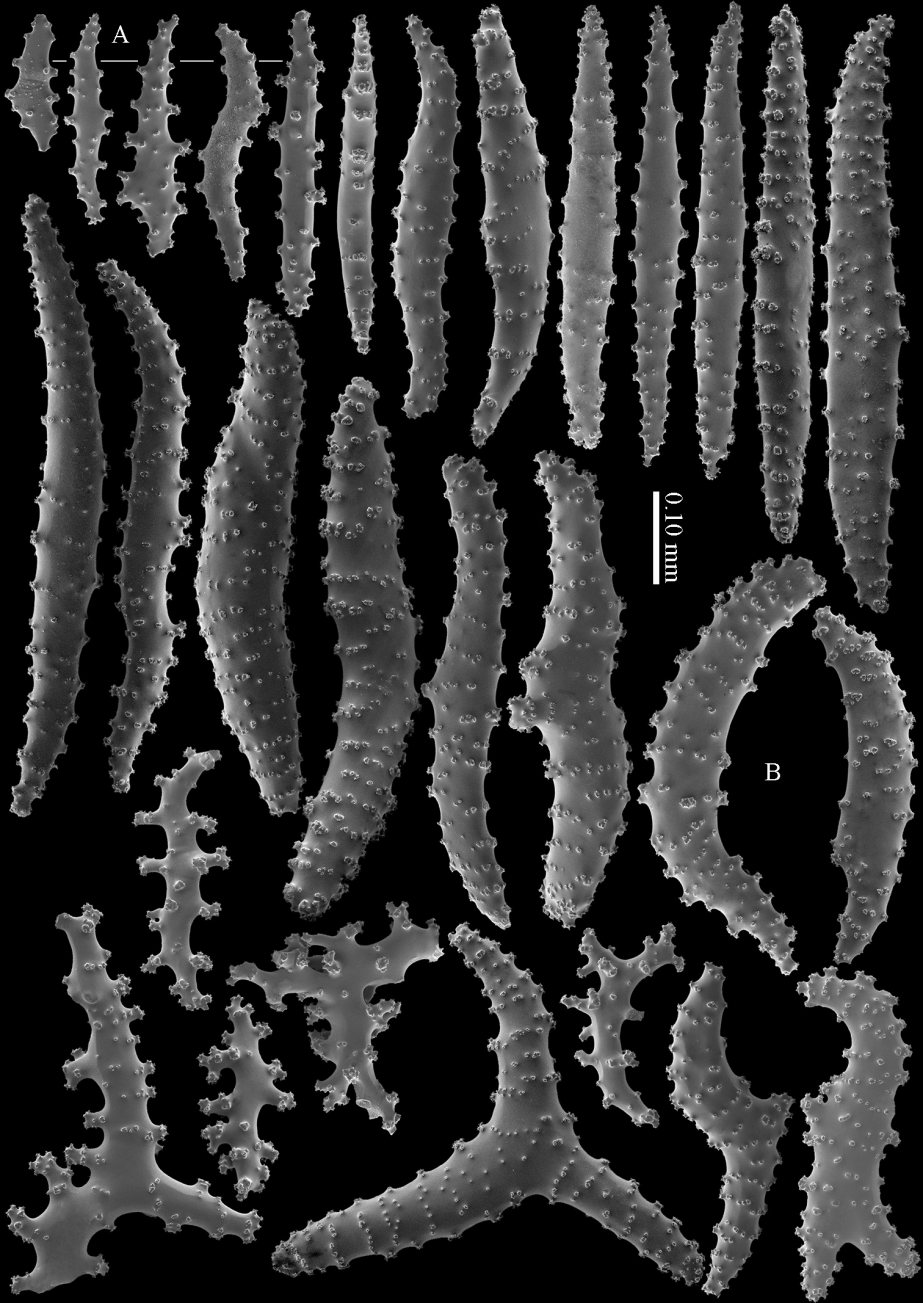
*Briareum violaceum* (Quoy & Gaimard, 1833), RMNH Coel. 41435; **A** sclerite of top calyx **B** sclerites of coenenchyme.

**Figure 22.**
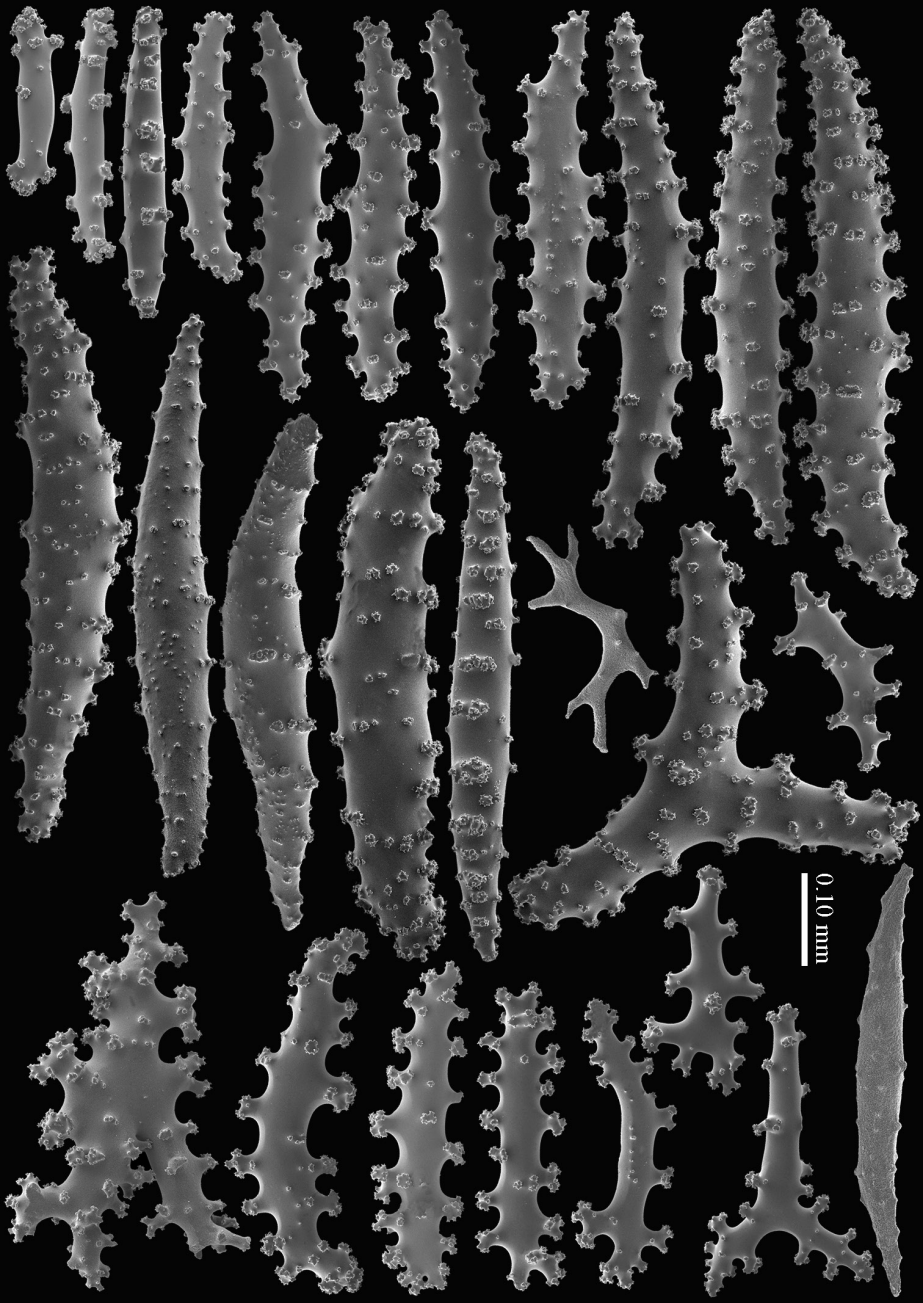
*Briareum violaceum* (Quoy & Gaimard, 1833), RMNH Coel. 41434, sclerites of coenenchyme.

**Figure 23.**
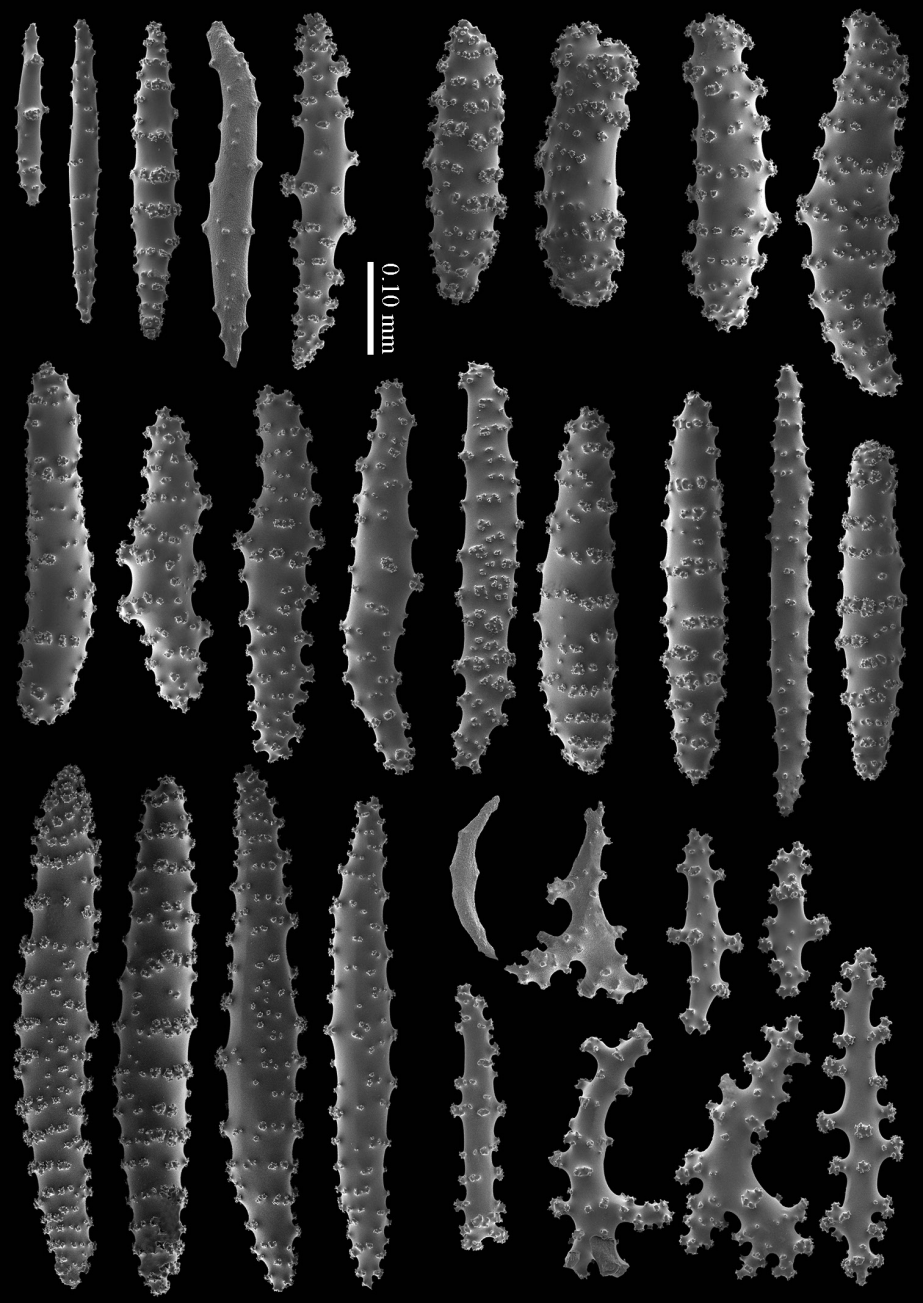
*Briareum violaceum* (Quoy & Gaimard, 1833), RMNH Coel. 41436, sclerites of coenenchyme.

RMNH Coel. 41434 (Figure 19C) and RMNH Coel. 41436 (Figure 19D) have unusually small spindles (Figs 22–23) approaching those found in *Briareum stechei*. This is probably due to the very short calyces in these specimens (Figs 19C–D).

**Figure 24.**
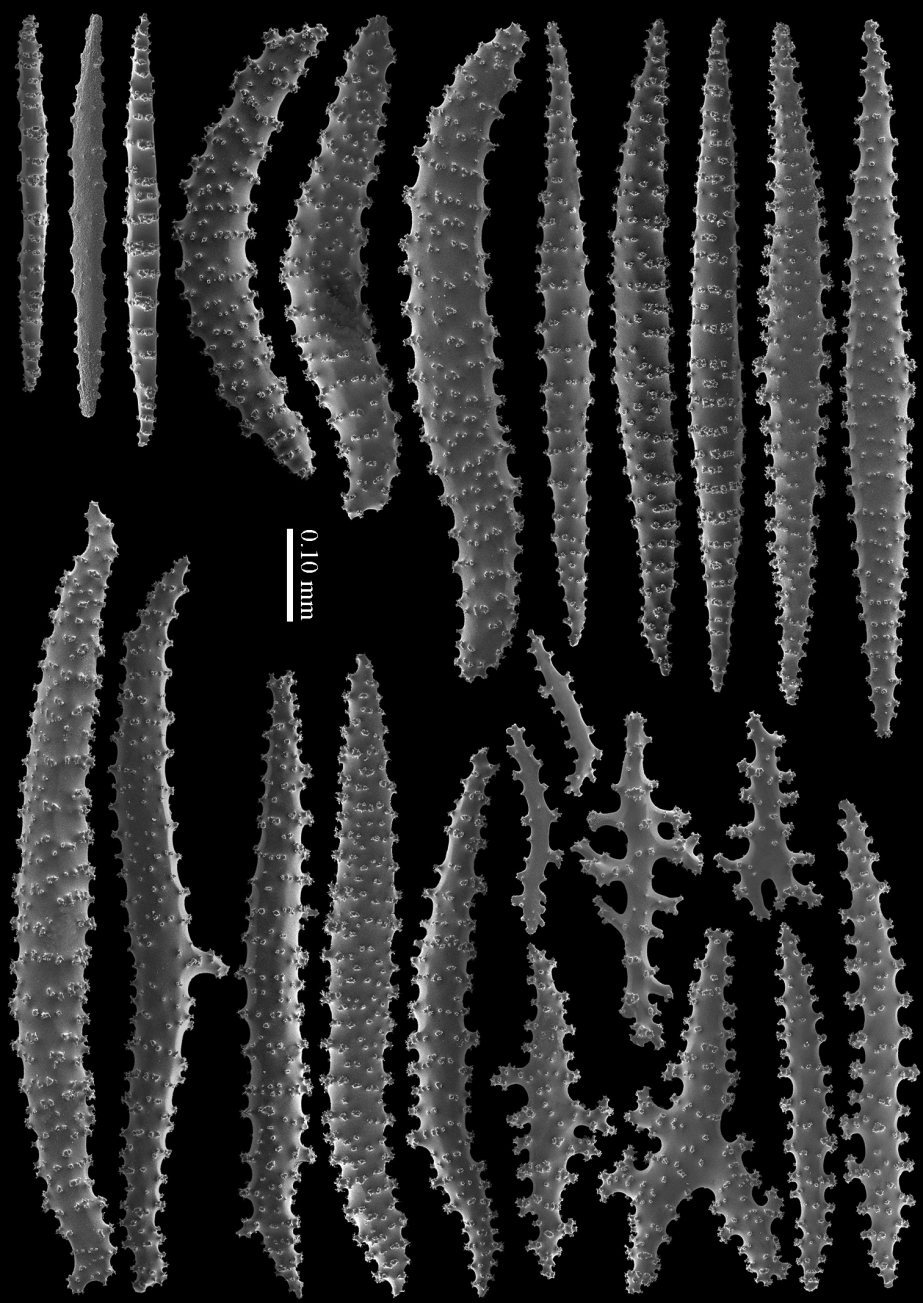
*Briareum violaceum* (Quoy & Gaimard, 1833), RMNH Coel. 41441, sclerites of coenenchyme.

**Figure 25.**
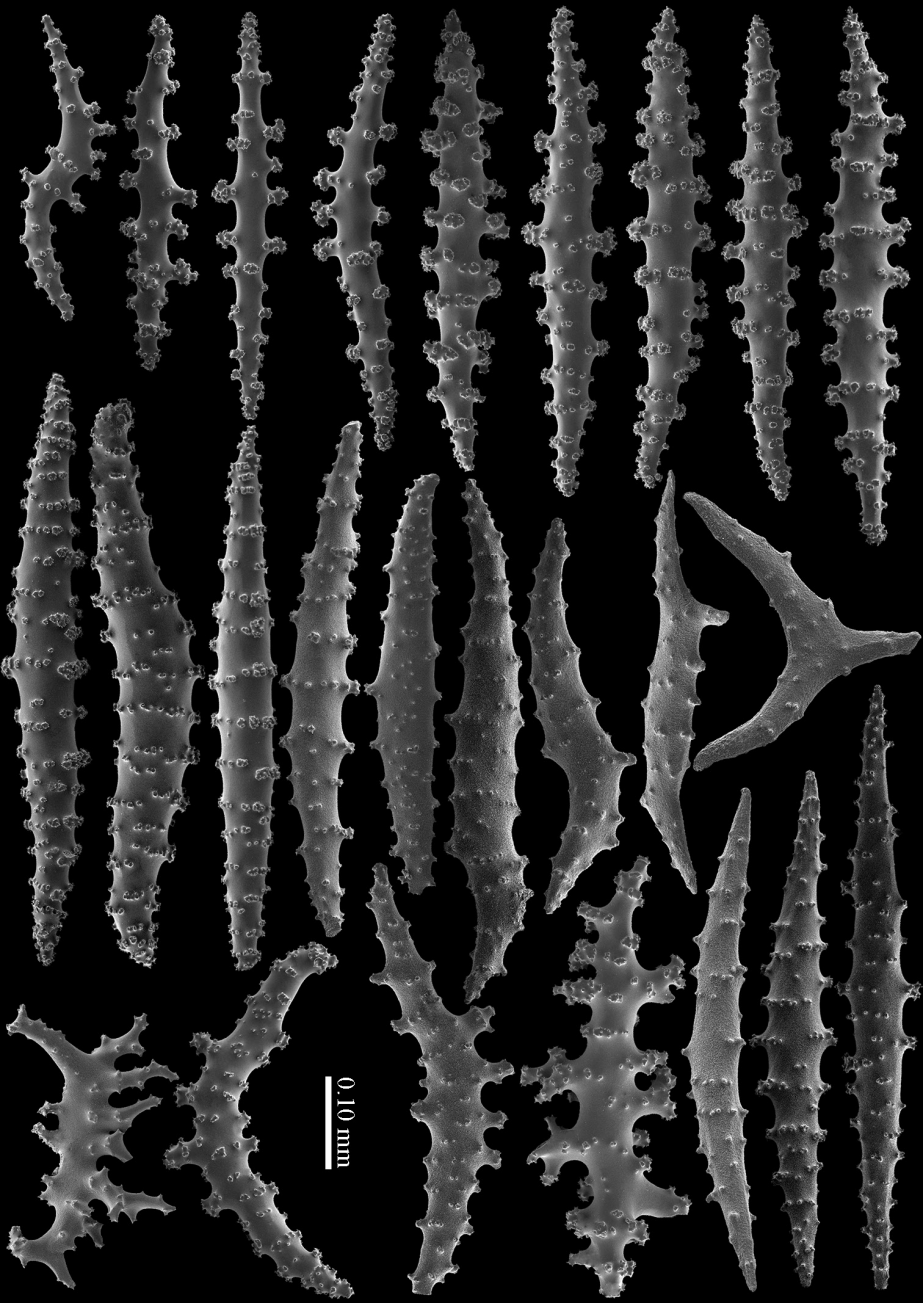
*Briareum violaceum* (Quoy & Gaimard, 1833), RMNH Coel. 41437, sclerites of coenenchyme.

Several specimens, RMNH Coel. 41441 (Figure 19E); RMNH Coel. 41439, RMNH Coel. 41437 (Figure 19F), RMNH Coel. 41438, and RMNH Coel. 41440 showed shorter more slender spindles, but with prominent tuberculation (Figs 24–25). RMNH Coel. 41441 (Figure 19E) has widely spaced big calyces; RMNH Coel. 41437 looks more like the type specimen. (Figure 19F). As all sclerites are magenta we provisionally include them in *Briareum violaceum*.

**Figure 26.**
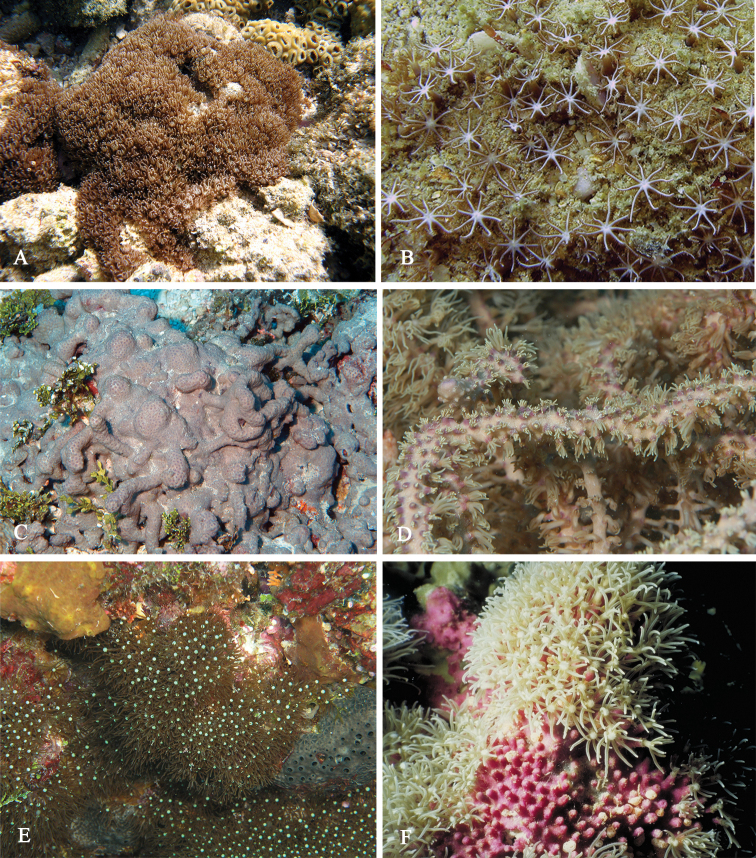
**A–B** *Briareum hamrum* (Gohar, 1948) **A** Colony **B** close-up of tentacles **C–D** *Briareum stechei* (Kükenthal, 1908) **C** Colony **D** close-up of tentacles **E–F** *Briareum violaceum* (Quoy & Gaimard, 1833) **E** Colony **F** close-up of tentacles.

#### Distribution.

Vanuatu, Japan (Ryukyu Archipelago, Bonin Islands), Taiwan, Coral Triangle, Australia (Great Barrier Reef).

## Discussion

All *Briareum* specimens deposited at the RMNH coelenterate collection were examined, from more than 50 localities around the world. The status of the Indo-Pacific *Briareum* species is reviewed and additional information provided. Moreover, a new taxon, *Briareum cylindrum* is described, and *Briareum excavatum* (Nutting, 1911) synonymised with *Briareum stechei* (Kükenthal, 1908). In total four *Briareum* species are recognized in the Indo-Pacific region; one recorded from the western Indo-Pacific, and the rest from the central and eastern Indo-Pacific.

The development in molecular and chemical studies, which reliably discriminate species, has been a challenge in cnidarians. Mitochondrial genes evolve slower than nuclear genes in anthozoans (Chen et al. 2009), therefore mitochondrial markers are invariant within and among genera (Shearer and Cofforth 2008). In octocorals, an extended mitochondrial barcode of COI plus the octocoral-specific mitochondrial gene mtMutS is usually diagnostic at the genus level and narrows species down to a small number of candidate sister taxa (McFadden et al. 2014). McFadden et al. (2011, 2014) included five *Briareum* specimens from Palau (RMNH Coel. 40883–40887) and one specimen from the Red Sea (ZMTAU CO34187) in their molecular studies using this marker. They distinguished three different species, two from Palau and one from the Red Sea. All Palau specimens were examined by us and proved to be indeed two species, *Briareum stechei* and *Briareum violaceum*. The one from the Red Sea identified by Prof. Benayahu represents *Briareum hamrum*. Miyazaki and Reimer (2014), who used other DNA markers and examined specimens from southern Japan, found three different morphological types of *Briareum* which seemed to be similar genetically and the authors suggested further analysis to reveal the phylogenetic relationships of these three types. Probably their material now can be identified with the morphological findings presented here.

This study shows variability in sclerite morphology among the examined material which is in agreement with the previous studies. Considering this fact, we decided not to complicate the situation with introducing more new species than necessary. Instead we grouped the species together based on major differences in sclerite shape and variability. Several examples in our examined specimens have somewhat different sclerite shapes, and they are considered as intraspecific variation.

Based on the examined underwater photographs, the polyp shape and colour pattern in the examined material of *Briareum hamrum* were consistent, having distinguishable pinnules with dark green to brown colour, white oral disk and white line along the tentacles (Figure 26A–B). The pinnules in this species were also noticed by Gohar (1948). In *Briareum stechei*, the pinnules were not distinguishable and in *Briareum violaceum* they were very small. There was no underwater *in situ* photograph of *Briareum cylindrum* available to us. These characters were not reported before, therefore their importance and consistency is yet to be understood.

*Briareum* shows a wide distribution range with one Atlantic and four Indo-West Pacific species. Our results showed that *Briareum hamrum* occurs only in the western and north-western Indian Ocean (Figure 1). This area consists of several sub-regions including East Africa, Seychelles, central Indian Ocean (Maldives and Chagos Archipelago), northwestern Indian Ocean (Arabian Sea, Oman Sea), Red Sea, and the Persian Gulf. The recent larval dispersal modelling suggests that the Red Sea and the Persian Gulf have the highest isolation in larval sources (Wood et al. 2014). This perhaps could explain the high number of endemic species described from these areas (Sheppard and Sheppard 1991; Sheppard et al. 2010; Samimi-Namin and van Ofwegen 2009), and suggests that the majority of the coral population maintained by high levels of self-seeding. *Briareum hamrum* clearly can tolerate high environmental fluctuations that exist in the Persian Gulf (Sheppard et al. 2010), and the Red Sea. *Briareum* species have not yet been recorded from the central Indian Ocean, Chagos Archipelago (Reinicke and van Ofwegen 1999), Maldives (Vennam and van Ofwegen 1996), and south west India (Herdman 1905; Thomson and Simpson 1909); however, it is expected to be found in these areas. The rest of the *Briareum* species have overlapping distribution in the central Indo-Pacific, which is expected due to its high levels of larval connectivity (Wood et al. 2014). More sampling efforts and examination of more material is necessary to clarify the distribution boundaries.

At present there are still uncertainties about the total number of *Briareum* species and their distribution boundaries, especially in the central Indo-Pacific. Further examination of newly collected material, together with *in situ* photographs (see e.g. Hoeksema and van Ofwegen 2004) and genetic material will eventually reveal the species characters and their variation along environmental gradients.

## Supplementary Material

XML Treatment for Briareum

XML Treatment for Briareum asbestinum

XML Treatment for Briareum cylindrum

XML Treatment for Briareum hamrum

XML Treatment for Briareum stechei

XML Treatment for Briareum violaceum
